# Age-related changes in *Drosophila* midgut are associated with PVF2, a PDGF/VEGF-like growth factor

**DOI:** 10.1111/j.1474-9726.2008.00380.x

**Published:** 2008-06

**Authors:** Na-Hyun Choi, Joong-Gook Kim, Dong-Jin Yang, Young-Shin Kim, Mi-Ae Yoo

**Affiliations:** 1 Department of Molecular Biology, College of Natural Science, Pusan National University Busan 609-735, South Korea; 2 Research Institute of Genetic Engineering, Pusan National University Busan 609-735, South Korea

**Keywords:** aging, Delta, *Drosophila* intestinal stem cell, oxidative stress, proliferative activity, PVF2

## Abstract

Age-associated changes in stem cell populations have been implicated in age-related diseases, including cancer. However, little is known about the underlying molecular mechanisms that link aging to the modulation of adult stem cell populations. *Drosophila* midgut is an excellent model system for the study of stem cell renewal and aging. Here we describe an age-related increase in the number and activity of intestinal stem cells (ISCs) and progenitor cells in *Drosophila* midgut. We determined that oxidative stress, induced by paraquat treatment or loss of catalase function, mimicked the changes associated with aging in the midgut. Furthermore, we discovered an age-related increase in the expression of PVF2, a *Drosophila* homologue of human PDGF/VEGF, which was associated with and required for the age-related changes in midgut ISCs and progenitor cell populations. Taken together, our findings suggest that PDGF/VEGF may play a central role in age-related changes in ISCs and progenitor cell populations, which may contribute to aging and the development of cancer stem cells.

## Introduction

Adult stem cells are tissue-specific cells that possess unique biological properties, including the ability to self-renew and to differentiate into all cell types of the tissue of origin (reviewed in Weissman, 2000). As a result of these properties, adult stem cells provide a continuous supply of differentiated cells to their tissue compartments, which is particularly important for homeostatic control in adult tissues. It has been hypothesized that the capacity of stem cells to maintain tissue homeostasis functionally declines with age, and that this decline may be responsible for many of the biological phenotypes associated with aging (reviewed in Van Zant & Liang, 2003). There is also a well-accepted hypothesis that aging creates conditions conducive to cancer development (reviewed in Bell & Van Zant, 2004; Balducci & Ershler, 2005; Krtolica, 2005). Therefore, an increased understanding of the age-related changes in stem cell populations should provide a platform for therapeutic applications to target age-related diseases. However, little is known about age-associated changes in adult stem cell populations.

The intestine is an excellent model system for the study of stem cell biology. Vertebrate and invertebrate intestines show marked similarities in terms of their development, cellular make-up and genetic control (Stainier, 2005). A recent study showed that proliferating progenitor cells reside within the midgut epithelium of adult *Drosophila*, similar to what has been observed in the vertebrate intestine (Micchelli & Perrimon, 2006; Ohlstein & Spradling, 2006). Adult *Drosophila* midgut cells can be separated into intestinal stem cells (ISCs), enteroblasts, enteroendocrine cells and enterocytes with specific markers. *Escargot*(*esg*), a transcription factor that belongs to the conserved Snail/Slug family, marks ISCs and enteroblasts within the adult *Drosophila* midgut (Micchelli & Perrimon, 2006). Prospero marks all known enteroendocrine cells (Micchelli & Perrimon, 2006; Ohlstein & Spradling, 2006). It was shown recently that ISCs could be identified by the expression of Delta, a ligand for the Notch receptor, and that the fates of ISC daughter cells are specified via differential Notch signaling with low or high levels of cytoplasmic Delta in ISCs (Ohlstein & Spradling, 2007). Therefore, *Drosophila* has emerged as a powerful model system for the elucidation of fundamental cellular pathways relevant to both intestinal stem cell biology and to aging.

Based on numerous studies involving humans and a variety of animal models, reactive oxygen species (ROS) are believed to contribute significantly to the aging process (reviewed in Dröge, 2003). Over time, the tissue environment accumulates cells that have undergone cellular senescence; these senescent cells secrete factors that may disrupt tissue architecture and stimulate neighboring cells to proliferate, thereby establishing a pro-oncogenic microenviroment (reviewed in Krtolica & Campisi, 2002). Vascular endothelial growth factor (VEGF) was initially identified as a tumor-secreted vascular permeability factor (Lobb *et al*., 1985). Since its discovery, overwhelming evidence has accumulated to support the role of VEGF as a principal regulator of tumor-associated angiogenesis and as an activator of tumor proliferation, invasion, and metastasis in mammals (reviewed in Dvorak, 2002; Hoeben *et al*., 2004). Previous studies have demonstrated that VEGF is expressed and secreted at low levels by most normal cells, whereas VEGF is constitutively and abundantly expressed by many human tumor cells, including colorectal carcinoma cells (Nakayama *et al*., 2002). The VEGF receptor flt-1 is located on intestinal epithelial cells (Siafakas *et al*., 1999); however, the functional role of VEGF in intestinal cells is not known. VEGF expression is affected by factors including hypoxia or nitric oxide, which can generate ROS (reviewed in Liu & Simon, 2004; Kuwabara *et al*., 2006). In *Drosophila*, three PDGF/VEGF homologues (PVF1, PVF2 and PVF3) and a single PDGF/VEGF receptor homologue (PVR) have been identified (Cho *et al*., 2002). PVF1 was reported to guide border cell migration in the egg chamber of *Drosophila* (McDonald *et al*., 2003). PVF2 is expressed in the developing gut during embryogenesis (Cho *et al*., 2002), and is involved in hemocyte proliferation (Munier *et al*., 2002). PVF3 is necessary for the proper organization of the wing disc epithelium by regulating the apical assembly of the actin cytoskeleton (Rosin *et al*., 2004).

In the current study, we investigated age-associated cellular changes in the adult *Drosophila* midgut. We observed age-related changes including an increase in the number of ISCs and a decrease in the number of differentiated enterocytes in the adult midgut. Furthermore, we determined that oxidative stress induced by paraquat treatment or loss of catalase function can induce phenotypic changes similar to those associated with aging. Finally, we demonstrated that these age- and oxidative stress-related phenotypes are correlated with increased PVF2 levels in the midgut, and that PVF2 function is required for the age- and oxidative stress-induced changes in midgut stem and progenitor cell populations. Our results provide insights into a novel PVF2-mediated mechanism for age-related modulation of intestinal stem populations and suggest that oxidative stress is likely to contribute significantly to age-related changes in intestinal stem cell biology.

## Results

### Age-related increases in the number of proliferating cells in adult *Drosophila* midgut

**Fig. 1:**
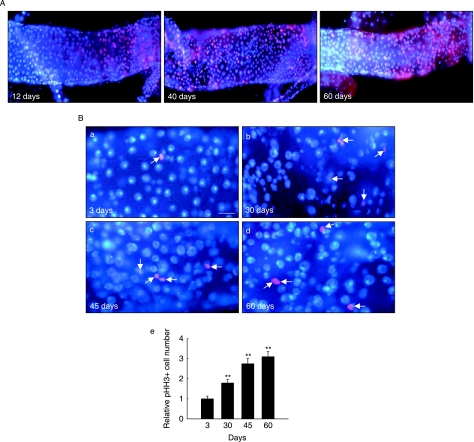
Age-related change in the number of proliferating cells in the adult midgut. (A) Age-related change in 5-bromodeoxyuridine (BrdU) incorporation levels in the adult midgut of wild-type flies. Two-, 30- and 50-day-old wild-type flies were fed on BrdU for 10 days, and stained with anti-BrdU. Overlay (DAPI, blue; anti-BrdU, red). Magnification is ×400. (B) Age-related change in the number of proliferating cells in the midgut. (a–d) The guts of 3-, 30-, 45- and 60-day-old wild-type flies were labeled with anti-phospho-histone H3 (arrow). Overlay (DAPI, blue; antiphospho-histone H3, red). Scale bar, 20 µm. (e) Relative number of the phospho-histone H3-positive cells detected per midgut of the 3-, 30-, 45- and 60-day-old wild-type flies. The number of phospho-histone H3-positive cells detected per midgut of 3-day-old flies was set at 1. Results are expressed as the mean ± SE values of 41–45 adults. Two asterisks represent *P* < 0.001 as compared to the 3-day-old flies.

To investigate age-related changes in *Drosophila* midgut, we first determined whether proliferative activity in the adult midgut is modulated with age. We compared the levels of DNA synthesis in the midguts of 12-, 40- and 60-day-old-flies using 5-bromodeoxyuridine (BrdU) labeling. We observed a striking and consistent age-related increase in BrdU-labeled cells in the adult midguts of the wild-type flies (Fig. 1A). Proliferating cell nuclear antigen (PCNA), which is important for G1-S phase progression (Duronio & O’Farrell, 1994), functions as a marker for cell proliferation (reviewed in Elias, 1997). In order to confirm the age-related increases in the proliferative activity of adult midgut cells, we evaluated the levels of PCNA mRNA in the midguts of 3-, 30- and 60-day-old wild-type flies. PCNA mRNA levels in the midgut of adult wild-type flies increased significantly with age (Supplementary Fig. S1A). We used an anti-phospho-histone H3 antibody to detect only the proliferating cells (Micchelli & Perrimon, 2006; Ohlstein & Spradling, 2006) among the ISCs and enteroblasts within the midgut of 3-, 30-, 45- and 60-day-old wild-type flies. Under these experimental conditions, we observed a significant increase in the number of phospho-histone H3-positive cells with age: 4.1 ± 0.6 in 3-day-old flies, 7.4 ± 0.7 in 30-day-old, 11.4 ± 1.1 in 45-day-old and 12.8 ± 1.1 in 60-day-old wild-type flies (Fig. 1B; Supplementary Table S1). These results showed that the number of proliferating cells in the adult *Drosophila* midgut increased with age.

### Age-related changes in the number and activity of ISCs and progenitor cells in *Drosophila* midgut

**Fig. 2:**
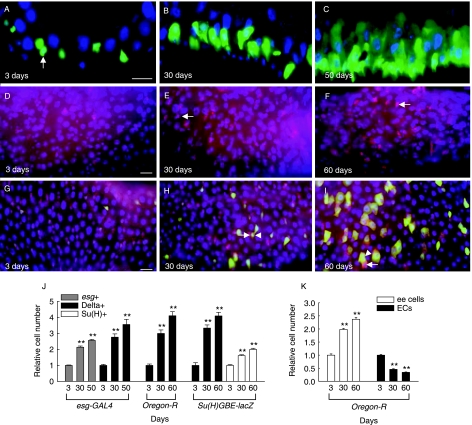
Age-related changes in the number of ISCs/and progenitor cells within the adult midgut. (A–C) Age-related change in the number of *esg*-positive cells (ISCs/or enteroblasts) in the adult midgut. Cross-sections of the posterior midguts of 3-, 30- and 50-day-old *esg-GAL4*,*UAS-GFP*/*CyO* flies were conducted with Cryostat and labeled with anti-GFP. Arrow indicates GFP-expressing cells under the control of *esg-GAL4*. Overlay (DAPI, blue; anti-GFP, green). (D–F) Age-related change in the number of Delta-positive cells (ISCs). The posterior midguts of 3-, 30- and 60-day-old wild-type flies were stained with anti-Delta (arrow). Overlay (DAPI, blue; anti-Delta, red). (G–I) Age-related change in the number of Su(H)-positive cells (enteroblasts). The posterior midguts of 3-, 30- and 60-day-old *Su(H)GBE-lacZ* flies were stained with anti-Delta (arrow) and anti-β-gal (arrowhead). Overlay (DAPI, blue; anti-β-gal, green; anti-Delta, red). All scale bar, 20 µm. (J) Graph showing age-related the relative numbers of *esg*-, Delta- or Su(H)-positive cells detected in the posterior midguts of *esg-GAL4*,*UAS-GFP*/*CyO*, wild-type and *Su(H)GBE-lacZ* flies. The cell numbers detected in the posterior midgut of 3-day-old flies were set at 1. For quantitative method of GFP-, Delta- and β-gal-expression in the posterior midgut, images were processed in Adobe Photoshop and then the cell number was counted in 0.06 × 0.02 cm area of the posterior midgut. The results are expressed as the mean ± SE values of 19–69 adults. (K) Graph showing age-related the relative numbers of enteroendocrine (ee) cells and enterocytes (ECs) with large nuclei detected in the posterior midgut of wild-type flies. The cell numbers detected in the posterior midgut of 3-day-old flies were set at 1. The midguts of 3-, 30- and 60-day-old wild-type flies were stained with anti-Prospero or DAPI, after which the numbers of enteroendocrine cells or enterocytes with large nuclei were assessed. For quantitative analysis, the images were processed in Adobe Photoshop and the number of enteroendocrine cells or enterocytes with large nuclei was counted in 0.12 × 0.02 cm or 0.06 × 0.02 cm area of the posterior midgut, respectively. The results are expressed as the mean ± SE values of 28–50 adults. All two asterisks represent *P* < 0.001 as compared to each 3-day-old flies.

We next assessed age-related changes in the number and activity of ISCs and progenitor cells by using specific intestinal cell markers. For this purpose, we first used *esg-GAL4*,*UAS-GFP*/*CyO* flies in which GFP is under the control of the *esg-GAL4* promoter and is expressed in *esg*-positive small cells, which correspond to ISCs or enteroblasts (Micchelli & Perrimon, 2006). The midguts of 3-, 30- and 50-day-old *esg-GAL4*,*UAS-GFP*/*CyO* flies were stained with an anti-GFP antibody. Interestingly, the number of GFP-positive cells increased significantly with age up to 2.6-fold in 50-day-old flies relative to 3-day-old flies (Fig. 2A–C,J; Supplementary Table S2). This result indicated that the number of ISCs and/or enteroblasts increased with age.

To determine whether the ISC numbers change with age, we examined the expression of Delta, which is a ligand for the Notch receptor and a highly specific stem cell marker, to identify ISCs (Ohlstein & Spradling, 2007). We assessed the number of Delta-positive cells within the midgut of the *esg-GAL4*,*UAS-GFP*/*CyO*, wild-type and *Su(H)GBE-lacZ* flies in which β-gal is expressed highly in enteroblasts from ISCs with high Delta levels and is weakly expressed in the enteroblasts from ISCs with low Delta levels (Ohlstein & Spradling, 2007). Interestingly, the number of Delta-positive cells increased significantly with age in *esg-GAL4*,*UAS-GFP*/*CyO*, wild-type and *Su(H)GBE-lacZ* flies (Fig. 2D–I,J, Table 1; Supplementary Table S3). The relative number of Delta-positive cells increased 3.6-fold in 50-day-old *esg-GAL4*,*UAS-GFP*/*CyO* flies, 4.1-fold in 60-day-old wild-type flies and 4.1-fold in 60-day-old *Su(H)GBE-lacZ* reporter flies relative to 3-day-old flies of each type (Fig. 2J; Supplementary Table S3). These results indicated that the number of ISCs within the *Drosophila* midgut increased with age. Analysis of Su(H)-positive cells indicated that the number of enteroblasts also increased with age (Fig. 2G–I,J; Supplementary Table S4). In addition, we determined the ratio of *esg*-positive/Delta-positive cells and Delta-positive/Su(H)-positive cells based on the number of *esg*-positive, Delta-positive and Su(H)-positive cells (Table 1); these results also indicated that ISC cell populations in the midgut increased with age.

**Table 1:** The changes in the ratio of *esg*-positive/Delta-positive cells and Delta-positive/Su(H)-positive cells with age, ROS production and PVF2/PVR overexpression

	Age (days)			ROS		PVF2/PVR		
			
	3	30	60	Control	Paraquat	Control	PVF2	PVR
*esg*-positive/Delta-positive	2.57	1.98	1.85	3.46	2.09	2.96	2.66	2.14
Delta-positive/Su(H)-positive	0.30	0.63	0.62	0.41	0.73	0.36	0.49	0.63

The ratios of *esg*-positive/Delta-positive cells with age and ROS production were based on the number of *esg*-positive and Delta-positive cells in *esg-GAL4*,*UAS-GFP*/*CyO* flies, and those by PVF2/PVR overexpression were based on the number of *esg*-positive and Delta-positive cells in *esg-GAL4*,*UAS-GFP*/+*, esg-GAL4*,*UAS-GFP*/*UAS-PVF2* and *esg-GAL4*,*UAS-GFP*/*UAS-PVR* flies (Supplementary Tables S2 and S3). The ratios of Delta-positive/Su(H)-positive cells with age and ROS production were based on the number of Delta-positive and Su(H)-positive cells in *Su(H)GBE-lacZ* flies, and those by PVF2/PVR overexpression were based on the number of Delta-positive and Su(H)-positive cells in *esg-GAL4*,*UAS-GFP*/+; *Su(H)GBE-lacZ*/+, *esg-GAL4*,*UAS-GFP*/*UAS-PVF2*; *Su(H)GBE-lacZ*/+ and *esg-GAL4*,*UAS-GFP*/*UAS-PVR*; *Su(H)GBE-lacZ*/+ flies (Supplementary Tables S3 and S4).

Individual ISCs have been shown to give rise to enteroblasts that become enteroendocrine cells and enterocytes (Ohlstein & Spradling, 2006). To determine the consequence of age-related ISC proliferation, we assessed the number of enteroendocrine cells and enterocytes. Prospero marks all known enteroendocrine cells within the adult *Drosophila* midgut (Micchelli & Perrimon, 2006; Ohlstein & Spradling, 2006). The number of enteroendocrine cells in the midgut increased in an age-dependent manner: 2.0-fold in 30-day-old flies and 2.4-fold in 60-day-old flies (Fig. 2K; Supplementary Table S5). In contrast to enteroendocrine cells, the number of differentiated enterocytes with large 4′,6-diamidino-2-phenylindole (DAPI)-labeled nuclei declined in an age-dependent manner: 0.5-fold in 30-day-old flies and 0.3-fold in 60-day-old flies (Fig. 2K; Supplementary Table S5). These findings suggested that the proper differentiation of enterocytes might be disrupted within the aged midgut. Taken together, these results demonstrated that the number and activity of ISCs and progenitor cells in the *Drosophila* midgut were subject to age-dependent modulation.

### Oxidative stress mimics age-related changes in *Drosophila* midgut cells

Because oxidative stress contributes significantly to age-related phenotypes, we determined whether oxidative stress could mimic the age-related changes in the adult midgut by using paraquat treatment and a catalase mutant (*Cat^n1^*). Paraquat treatment, which is known to induce toxicity in cells by stimulating oxygen utilization via redox-cycling coupled with the generation of reactive oxygen intermediates (reviewed in Bus & Gibson, 1984), has been employed successfully to induce oxidative stress in a variety of organisms (Monnier *et al*., 2002; Fujii *et al*., 2004). First, we established a working paraquat dose and treatment time (10 mm, 16 h) that induced an intermediate stress response, but did not affect survival rates. The *Cat^n1^* mutant flies showed a gene dosage-dependent effect on catalase activity and a decreased lifespan (Griswold *et al*., 1993).

**Fig. 3:**
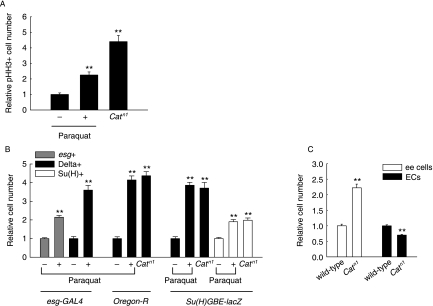
Oxidative stress-induced changes in the number of ISCs and progenitor cells within the adult midgut. (A) Effects of paraquat treatment and the *Cat^n1^* allele on the number of phospho-histone H3-positive cells. The midguts of 5-day-old wild-type flies exposed to 10 mm paraquat in 1% sucrose or 1% sucrose media as controls for 16 h, and 5-day-old *Cat^n1^* mutant or wild-type flies as controls were labeled with anti-phospho-histone H3. Graph shows the relative number of phospho-histone H3-positive cells per midgut. The number of phospho-histone H3-positive cells observed per midgut of wild-type flies was set at 1. Results are expressed as the mean ± SE values of 39–52 adults. (B) Effects of paraquat treatment and the *Cat^n1^* allele on the number of ISCs and progenitor cells in the adult midgut. The midguts of 5-day-old *esg-GAL4*,*UAS-GFP*/*CyO*, wild-type and *Su(H)GBE-lacZ* flies exposed to 10 mm paraquat in 1% sucrose or 1% sucrose media as control for 16 h, 5-day-old wild-type, *Cat^n1^* mutant, *Su(H)GBE-lacZ*/+ and *Su(H)GBE-lacZ*/*Cat^n1^* flies were labeled with anti-GFP, anti-Delta and anti-β-gal. Graph shows the relative numbers of *esg*-, Delta- or Su(H)-positive cells in the posterior midgut. The cell numbers detected in the posterior midgut of paraquat-untreated flies, wild-type and *Su(H)GBE-lacZ*/+ flies were set at 1, respectively. For quantitative method of GFP-, Delta- and β-gal-expression in the posterior midgut, images were processed in Adobe Photoshop and then the cell number was counted in 0.06 × 0.02 cm area of the posterior midgut. The results are expressed as the mean ± SE values of 16–35 adults. (C) Effects of *Cat^n1^* allele on the number of enteroendocrine cells and enterocytes with large nuclei in the adult midgut. Graph shows the relative numbers of enteroendocrine (ee) cells and enterocytes (ECs) in the adult posterior midguts of *Cat^n1^* mutant flies as compared to wild-type flies. The cell numbers detected in the posterior midgut of wild-type flies were set at 1. The midguts of 5-day-old wild-type and *Cat^n1^* mutant flies were stained with anti-Prospero or DAPI, after which the numbers of enteroendocrine cells or enterocytes with large nuclei were assessed. For quantitative analysis, the images were processed in Adobe Photoshop and then the number of enteroendocrine cells or enterocytes with large nuclei was counted in 0.12 × 0.02 cm or 0.06 × 0.02 cm area of the posterior midgut, respectively. The results are expressed as the mean ± SE values of 31–40 adults. All two asterisks represent *P* < 0.001 as compared to each control.

In order to confirm that the change in the redox balance occurred in the midgut of flies harboring the *Cat^n1^* amorphic allele, ROS generation in the midguts of the *Cat^n1^* mutant flies was compared to that of wild-type flies. ROS generation in the midguts of the *Cat^n1^* mutant female and male flies was significantly higher than that in wild-type flies (Supplementary Fig. S2). Using these experimental conditions, the effects of paraquat treatment and the *Cat^n1^* allele on DNA synthesis in the midgut were analyzed using BrdU labeling. In both paraquat-treated and *Cat^n1^* mutant flies, we detected a marked increase in the number of BrdU-labeled cells (Supplementary Fig. S3A). Paraquat treatment and the *Cat^n1^* mutation also induced an increase in PCNA mRNA levels in the adult midgut, as determined by reverse transcription–polymerase chain reaction (RT-PCR) (Supplementary Fig. S1A). We also assessed the effect of oxidative stress on the number of proliferating cells in the adult midgut using anti-phospho-histone H3 antibody staining. The number of cells labeled with anti-phospho-histone H3 antibody increased significantly in response to oxidative stress: 9.0 ± 0.8 in the paraquat-treated flies and 17.6 ± 1.6 in the *Cat^n1^* mutant flies compared to 3.9 ± 0.4 and 3.9 ± 0.5 in control flies, respectively (Fig. 3A; Supplementary Fig. S3B and Table S1). These results showed that the number of proliferating cells in the adult midgut increased in response to oxidative stress in a manner similar to that observed in aging flies.

We then determined whether oxidative stress also modulated the number and activity of ISCs and progenitor cells of the adult midgut. First, the number of *esg*-positive cells increased 2.1-fold in paraquat-treated midguts of *esg-GAL4*,*UAS-GFP*/*CyO* adult flies as compared to untreated control flies (Fig. 3B; Supplementary Table S2). This result indicated that the number of ISCs or enteroblasts could be modulated by oxidative stress. We then determined whether the number of ISCs changed in response to oxidative stress. The number of Delta-positive cells increased 3.6-fold, 4.1-fold and 4.4-fold in the adult midgut of paraquat-treated *esg-GAL4*,*UAS-GFP*/*CyO* flies, paraquat-treated wild-type flies and *Cat^n1^* mutant flies relative to negative controls, respectively (Fig. 3B, Table 1; Supplementary Fig. S4a–c and Table S3). In addition, the midgut from paraquat-treated *Su(H)GBE-lacZ* flies and *Cat^n1^*/*Su(H)GBE-lacZ* flies also showed increased numbers of ISCs: 3.9-fold and 3.7-fold relative to negative controls, respectively (Fig. 3B; Supplementary Fig. S4d–f and Table S3). The midgut from paraquat-treated *Su(H)GBE-lacZ* flies and *Cat^n1^*/*Su(H)GBE-lacZ* flies showed increased the number of Su(H)-positive cells: 1.9-fold and 2.0-fold relative to negative controls, respectively (Fig. 3B; Supplementary Fig. S4d–f and Table S4). The ratios of *esg*-positive/Delta-positive cells and Delta-positive/Su(H)-positive cells in the midguts from paraquat-treated *esg-GLA4*,*UAS-GFP*/*CyO* and *Su(H)GBE-lacZ* flies were also compared to those of control flies (Table 1). These results showed an increase in ISC populations in response to oxidative stress. To determine the consequence of having an increased number of proliferating cells following oxidative stress, the number of enteroendocrine cells and enterocytes of *Cat^n1^* mutant flies were compared with those of wild-type flies. Whereas the number of enteroendocrine cells increased by 2.2-fold, the number of differentiated enterocytes with large nuclei was reduced by 0.7-fold in the adult midgut of *Cat^n1^* mutant flies relative to wild-type flies; this finding was similar to that associated with aging (Fig. 3C; Supplementary Table S5). Collectively, these results demonstrate that oxidative stress affects the number and activity of stem cells and progenitor cells in the *Drosophila* midgut in a manner similar to that associated with normal aging.

### Expression of the *Pvf2-lacZ* reporter in ISCs and enteroblasts of the adult *Drosophila* midgut

Over time, the tissue environment accumulates cells that have undergone cellular senescence, and these senescent cells secrete factors that may disrupt tissue architecture and induce proliferation in neighboring cells, thereby creating a pro-oncogenic microenviroment (reviewed in Krtolica & Campisi, 2002). In mammals, VEGF is a principal regulator of tumor-associated angiogenesis and an activator of tumor cell proliferation, invasion, and metastasis (reviewed in Dvorak, 2002; Hoeben *et al*., 2004). VEGF regulates hematopoietic stem cell proliferation via an autocrine loop mechanism (reviewed in Gerber & Ferrara, 2003; Fragoso *et al*., 2007), and the VEGF receptor is expressed on intestinal epithelial cells (Siafakas *et al*., 1999). PVF2, a *Drosophila* homologue of PDGF/VEGF, is involved in hemocyte proliferation in *Drosophila* larvae (Munier *et al*., 2002) and is expressed in the developing gut during embryogenesis (Cho *et al*., 2002).

**Fig. 4:**
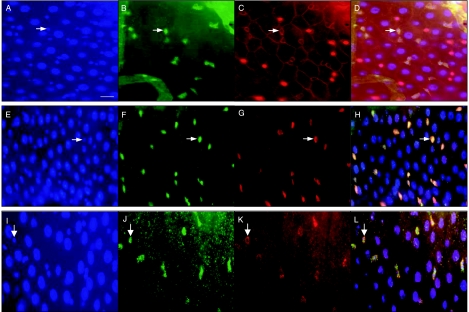
Expression of the *Pvf2-lacZ* reporter gene in the ISCs/or enteroblasts of the adult midgut. (A–D) *Pvf2-lacZ* expression was detected in the Prospero-negative small cells in the adult midgut including two copies in the *Pvf2-lacZ* flies. a, DAPI (blue). B, anti-β-gal (green). C, anti-Prospero and anti-Armadillo (red). D, Overlay. Enteroendocrine cells (arrowhead) are marked with nuclear Prospero (A, C and D). All cells were outlined with membrane-enriched Armadillo (C and D). Prospero-negative and *Pvf2-lacZ*- positive small cells were marked with arrows. (E–H) *Pvf2-lacZ* expression is co-localized with *esg*-GFP, a marker of ISCs/or enteroblasts, in the adult midgut of *esg-GAL4*,*UAS-GFP*/*Pvf2-lacZ* flies. E, DAPI (blue). F, anti-GFP (green). G, antiβ-gal (red). H, Overlay. *Pvf2-lacZ*- expression in *esg*-positive small cells is indicated with an arrow. (I–L) *Pvf2-lacZ* expression is colocalized with Delta-positive cells, a marker for ISCs, in the adult midgut. I, DAPI (blue). J, anti-β-gal (green). K, anti-Delta (red). L, Overlay. *Pvf2-lacZ* expression in Delta-positive cells is indicated with an arrow. Scale bar, 20 µm.

We hypothesized that PVF2 may modulate age-related changes in the adult fly midgut. To test this hypothesis, we first determined whether *Pvf2* expression is modulated by age and by oxidative stress. For this purpose, we initially established *Pvf2* transgenic reporter flies containing the *Pvf2* promoter region (–1220 to +281 with respect to the transcription initiation site) fused to *lacZ*. Consistent with the results of a previous study in which the *Pvf2* gene was found to be expressed in the gut during embryonic development (Cho *et al*., 2002), we detected abundant expression of the *Pvf2-lacZ* reporter in the fly foregut and hindgut during early developmental stages and throughout the entire gut during late developmental stages (Supplementary Fig. S5a). *Pvf2-lacZ* expression was also observed in the whole gut of third-instar larvae (Supplementary Fig. S5b). As the expression of the *Pvf2-lacZ* reporter was detected in specific cell populations of the adult midgut, we determined the adult midgut cell types that expressed *Pvf2-lacZ*. We carried out immunostaining experiments using anti-β-gal, anti-Prospero, and anti-Armadillo antibodies. All cells within the adult *Drosophila* midgut epithelium are outlined with membrane-enriched Armadillo staining (Ohlstein & Spradling, 2006). Interestingly, *Pvf2-lacZ* reporter expression was detected only in the Prospero-negative small cells, which represent a population of ISCs and enteroblasts (Micchelli & Perrimon, 2006) (Fig. 4A–D). In order to further characterize the expression of the *Pvf2-lacZ* reporter in ISCs and enteroblasts, we utilized *esg-GAL4*,*UAS-GFP*/*CyO* flies. We found that *Pvf2-lacZ* expression co-localized specifically with *esg*-GFP expression in the midgut cells (Fig. 4E–H), suggesting ISC and/or enteroblast expression. In addition, the ratio of β-gal-positive/*esg*-positive cells in *Pvf2-lacZ*/*esg-GAL4*,*UAS-GFP* flies was approximately ‘1’ (Supplementary Table S6). In order to confirm the expression of *Pvf2-lacZ* in ISCs, we stained the adult midgut cells of *Pvf2-lacZ* reporter flies with anti-β-gal and anti-Delta antibodies. We found that *Pvf2-lacZ* was expressed in Delta-positive cells, indicating that some of the *Pvf2-lacZ*-positive cells corresponded to ISCs (Fig. 4I–L). Collectively, these results showed that the *Pvf2-lacZ* fusion gene was expressed specifically in ISCs and enteroblasts of the adult *Drosophila* midgut.

### Age- and oxidative stress-induced increases in *Pvf2* expression in the adult midgut

**Fig. 5:**
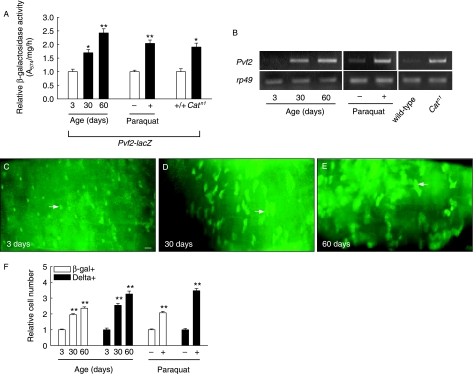
Increase of *Pvf2* level in the adult midgut with age and by oxidative stress. (A) Effects of aging or oxidative stress on the β-galactosidase activity in the midgut of flies including two copies of the *Pvf2-lacZ*. The β-galactosidase activities in the midguts of 3-, 30- and 60-day-old *Pvf2-lacZ* flies, 5-day-old *Pvf2-lacZ* flies exposed to 10 mm paraquat in 1% sucrose or 1% sucrose media as a control for 16 h, 5-day-old *Pvf2-lacZ*/+;+/+ and *Pvf2-lacZ*/+;*Cat^n1^*/+ flies were examined. Relative levels of β-galactosidase activity are expressed as the mean ± SE values of three independent experiments with three replicates per experiment. Two asterisks represent *P* < 0.001, and a single asterisk represents *P* < 0.002 as compared to each control. (B) *Pvf* 2 mRNA levels in the midgut of aged, paraquat-treated, and *Cat^n1^* mutant flies. Total RNA was isolated from the midguts of 3-, 30- and 60-day-old wild-type flies, 5-day-old wild-type flies exposed to 10 mm paraquat in 1% sucrose or 1% sucrose media as controls for 16 h, 5-day-old wild-type and *Cat^n1^* mutant flies. Then cDNAs were synthesized and *Pvf2* mRNA levels were analyzed via RT-PCR. *rp49* was used as a loading control. (C–E) Age-related change in the number of *Pvf2-lacZ*-positive cells within the adult midgut. Anti-β-gal expression (green, arrow) of the midgut of 3-, 30- and 60-day-old flies including two copies of the *Pvf2-lacZ* was examined. Scale bar, 20 µm. (F) Graph showing age-or oxidative stress-related the relative numbers of *Pvf2-lacZ*-positive cells and Delta-positive cells detected in the posterior midgut of *Pvf2-lacZ* flies. The midguts of 3-, 30- and 60-day-old *Pvf2-lacZ* flies, 5-day-old *Pvf2-lacZ* flies exposed to 10 mm paraquat in 1% sucrose or 1% sucrose media as controls for 16 h were labeled with anti-β-gal and anti-Delta. The cell numbers detected in the posterior midgut of 3-day-old or paraquat untreated *Pvf2-lacZ* flies were set at 1. For quantitative method of β-gal- and Delta-expression in the posterior midgut, images were processed in Adobe Photoshop and then the cell number was counted in 0.06 × 0.02 cm area of the posterior midgut. The results are expressed as the mean ± SE values of 27–40 adults. Two asterisks represent *P* < 0.001 as compared to each control.

Because *Pvf2-lacZ* is expressed in ISCs and enteroblasts, we investigated whether *Pvf2* levels in the adult midgut increase with age and in response to oxidative stress. First, quantitative measurements of β-galactosidase activities in the midguts of 3-, 30- and 60-day-old *Pvf2-lacZ* flies showed a marked increase with age (Fig. 5A). On average, we noted a 1.7-fold and 2.4-fold increase in *Pvf2-lacZ* expression in the guts of the 30-day-old and 60-day-old flies relative to 3-day-old flies, respectively. In order to determine whether *Pvf2* levels in the adult midgut are also affected by oxidative stress, the effects of paraquat treatment on *Pvf2* levels in the adult midgut were determined. β-Galactosidase activity in the midguts of *Pvf2-lacZ* reporter flies treated with paraquat increased approximately 2.0-fold relative to control flies (Fig. 5A). The effect of the *Cat^n1^* mutant allele on *Pvf2* levels in the midgut was also assessed. β-Galactosidase activity in the midguts of 5-day-old *Pvf2-lacZ*/+; *Cat^n1^*/+ flies was approximately 1.9-fold higher than that of the *Pvf2-lacZ*/+;+/+ flies (Fig. 5A). In order to confirm the age-related increase in the *Pvf2* production in the midgut, we assessed *Pvf2* mRNA levels within the midguts of 3-, 30- and 60-day-old flies using RT-PCR. The levels of *Pvf2* mRNA in the midgut increased significantly with age (Fig. 5B). The *Pvf2* mRNA levels in the midgut of paraquat-treated and *Cat^n1^* mutant flies also increased relative to negative controls (Fig. 5B). Taken together, our results indicate that *Pvf2* expression in the adult midgut increases with age and in response to oxidative stress.

To determine whether the age-related increase in β-galactosidase activity associated with the *Pvf2-lacZ* gene is due to an age-dependent increase in ISC and enteroblast cell populations, age-related changes in the number of *Pvf2-lacZ*-positive cells in the adult midgut were evaluated. The number of *Pvf2-lacZ*-positive cells in the adult midgut was 2.0-fold higher in 30-day-old flies and 2.4-fold higher in 60-day-old flies relative to 3-day-old flies, respectively (Fig. 5C–F; Supplementary Table S7). The number of *Pvf2-lacZ*-positive cells in the paraquat-treated midgut also increased 2.1-fold relative to the untreated controls (Fig. 5F; Supplementary Table S7). In addition, the number of Delta-positive cells in the aged or paraquat-treated midgut of *Pvf2-lacZ* flies was compared to that of control flies. The number of Delta-positive cells increased 2.6-fold in 30-day-old flies and 3.3-fold in 60-day-old flies relative to 3-day-old flies (Fig. 5F; Supplementary Table S7). The number of Delta-positive cells in paraquat-treated midgut increased 3.5-fold relative to untreated flies (Fig. 5F; Supplementary Table S7). Based on the number of *Pvf2-lacZ*-positive cells or Delta-positive cells in *Pvf2-lacZ* flies, the ratio of β-gal-positive/Delta-positive cells was also determined (Supplementary Table S7): a reduction in the ratio of β-gal-positive/Delta-positive cells was detected in aged and paraquat-treated flies. These results suggested that the ISC populations in the midgut of the *Pvf2-lacZ* flies increased with aging and in response to paraquat treatment; a similar increase in ISC populations was observed in *esg-GAL4,UAS-GFP*/*CyO* flies (Table 1). Collectively, these results indicated that age- or oxidative stress-related increases in PVF2 expression in the midgut may be due to an increase in the number of ISCs and enteroblasts which produce PVF2 in the midgut.

### The role of PVF2 with respect to the number and activity of stem and progenitor cells in *Drosophila* midgut

Because PVF2 levels in the adult fly midgut increased with age and in response to oxidative stress, we investigated the functional role of PVF2 with respect to the number and activity of stem and progenitor cells in the midgut by using a *Pvf2* mutant allele, *Pvf2^c06947^*, which includes a homozygous viable *piggyback*[w+] insertion within the *Pvf2* gene (Cho *et al*., 2002), a fly strain containing *UAS-PVF2-RNAi*, which targets PVF2 expression in the presence of GAL4, a fly strain containing *UAS-PVF2* (Cho *et al*., 2002), which overexpresses PVF2 in the presence of GAL4, a fly strain containing *UAS-PVR* (Duchek *et al*., 2001), which overexpresses the PDGF/VEGF receptor homologue PVR in the presence of GAL4, and the *esg-GAL4* flies, which expresses GAL4 in midgut ISCs and enteroblasts. When *UAS-PVF2*/*UAS-PVF2*, *UAS-PVR*/*UAS-PVR* or *UAS-PVF2-RNAi*/*CyO* flies were each crossed with *esg-GAL4,UAS-GFP/CyO*, only a small fraction of the *esg-GAL4,UAS-GFP*/*UAS-PVF2* and *esg-GAL4,UAS-GFP*/*UAS-PVR* flies developed into adults (Supplementary Table S8). The relatively small number of *esg-GAL4,UAS-GFP*/*UAS-PVF2* and *esg-GAL4,UAS-GFP*/*UAS-PVR* adults that survived suggests that *esg-GAL4*-induced overexpression of PVF2 or PVR may have deleterious effects during development.

**Fig. 6:**
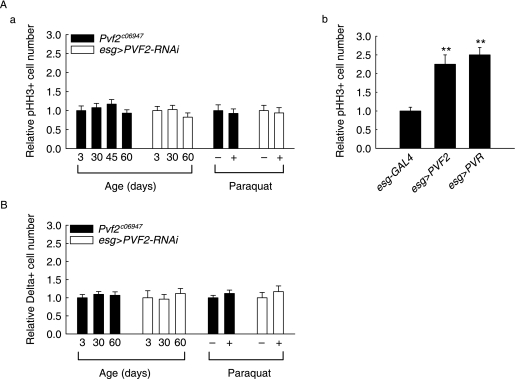

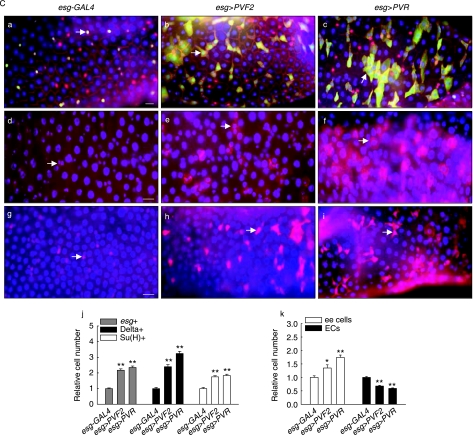
Effects of PVF2 on the number and activity of ISCs and progenitor cells in the adult midgut. (A) Effects of PVF2 level on the number of proliferating cells in the adult midgut. (a) Effects of aging or oxidative stress on the number of proliferating cells in the adult midgut of *Pvf2^c06947^* mutant or PVF2 knockdown flies. The midguts of 3-, 30-, 45- and 60-day-old *Pvf2^c06947^* flies and 3-, 30-and 60-day-old the *esg-GAL4*/+;*UAS-PVF2-RNAi*/+ flies were labeled with anti-phospho-histone H3. The midguts of 5-day-old *Pvf2^c06947^* and *esg-GAL4*/+;*UAS-PVF2-RNAi*/+ flies exposed to 10 mm paraquat in 1% sucrose or 1% sucrose media as a control for 16 h were labeled with anti-phospho-histone H3. The relative number of the phospho-histone H3-positive cells detected per midgut was shown. The number of phospho-histone H3-positive cells detected per midgut of 3-day-old or paraquat-untreated flies was set at 1. Results are expressed as the mean ± SE values of 28–61 adults. (b) Effect of PVF2 or PVR overexpression under *esg-GAL4* on the number of proliferating cells in the adult midgut. The midguts of 3-day-old *esg-GAL4*,*UAS-GFP*/+, *esg-GAL4*,*UAS-GFP*/*UAS-PVF2* and *esg-GAL4*,*UAS-GFP*/*UAS-PVR* were labeled with antiphospho-histone H3. The relative number of the phospho-histone H3-positive cells detected per midgut was shown. The number of phospho-histone H3-positive cells detected per midgut of 3-day-old *esg-GAL4*,*UAS-GFP*/+ flies was set at 1. Results are expressed as the mean ± SE values of 52–56 adults. Two asterisks represent *P* < 0.001 as compared to control. (B) Effect of aging or oxidative stress on the number of Delta-positive cells in the adult midgut of *Pvf2^c06947^* mutant or PVF2 knockdown flies. The midguts of 3-, 30- and 60-day-old *Pvf2^c06947^* and *esg-GAL4*/+;*UAS-PVF2-RNAi*/+ flies were labeled with anti-Delta. The midguts of 5-day-old *Pvf2^c06947^* and *esg-GAL4*/+;*UAS-PVF2-RNAi*/+ flies exposed to 10 mm paraquat in 1% sucrose or 1% sucrose media as a control for 16 h were labeled with anti-Delta. The relative number of the Delta-positive cells detected in the posterior midgut was shown. The cell number detected in the posterior midgut of 3-day-old or paraquat-untreated flies was set at 1. For quantitative method of Delta-expression in the posterior midgut, images were processed in Adobe Photoshop and then the cell number was counted in 0.06 × 0.02 cm area of the posterior midgut. Results are expressed as the mean ± SE values of 17–32 adults. (C) Effect of PVF2 or PVR overexpression under *esg-GAL4* on the number of ISCs and progenitor cells in the adult midgut. (a–c) Effect of PVF2 or PVR overexpression under *esg-GAL4* on the number of *esg*-positive cells in the adult midgut. The midguts of the 3-day-old *esg-GAL4*,*UAS-GFP/+*(a), *esg-GAL4*,*UAS-GFP*/*UAS-PVF2*(b) and *esg-GAL4*,*UAS-GFP*/*UAS-PVR*(c) flies were stained with anti-GFP (arrow), anti-Armadillo and anti-Prospero. Overlay (DAPI, blue; anti-GFP, green; anti-Armadillo and anti-Prospero, red). (d–f) Effect of PVF2 or PVR overexpression under *esg-GAL4* on the number of Delta-positive cells in the adult midgut. The midguts of the 3-day-old *esg-GAL4*,*UAS-GFP/+*(d), *esg-GAL4*,*UAS-GFP*/*UAS-PVF2*(e) and *esg-GAL4*,*UAS-GFP*/*UAS-PVR*(g) flies were stained with anti-Delta (arrow). Overlay (DAPI, blue; anti-Delta, red). (g–i) Effect of PVF2 or PVR overexpression under *esg-GAL4* on the number of Su(H)-positive cells in the adult midgut. The adult midguts of 3-day-old *esg-GAL4*,*UAS-GFP*/+;*Su(H)GBE-lacZ*/+ (g), *esg-GAL4*,*UAS-GFP*/*UAS-PVF2*; *Su(H)GBE-lacZ*/+ (h) and *esg-GAL4*,*UAS-GFP*/*UAS-PVR*;*Su(H)GBE-lacZ*/+ (i) flies were stained with anti-β-gal (arrow). Overlay (DAPI, blue; antiβ-gal, red). All scale bar, 20 µm. (j) Graph showing the relative numbers of *esg*-, Delta- or Su(H)-positive cells in the adult midgut of flies containing both *UAS-PVF2* and *esg-GAL4* or *UAS-PVR* and *esg-GAL4*. The cell numbers in the midgut of 3-day-old *esg-GAL4*,*UAS-GFP/+* or *esg-GAL4*,*UAS-GFP*/+;*Su(H)GBE-lacZ*/+ flies were set at 1. For quantitative analysis, the images were processed in Adobe Photoshop and the cell number was counted in 0.06 × 0.02 cm area of the posterior midgut. The results are expressed as the mean ± SE values of 25–46 adults. Two asterisks represent *P* < 0.001 as compared to each control. (k) Effects of PVF2 or PVR overexpression under *esg-GAL4* on the number of enteroendocrine cells and enterocytes with large nuclei. Graph shows the relative numbers of enteroendocrine (ee) cells and enterocytes (ECs) with large nuclei detected in the posterior midguts of 3-day-old *esg-GAL4*,*UAS-GFP*/*UAS-PVF2* and *esg-GAL4*,*UAS-GFP*/*UAS-PVR* flies. The cell numbers detected in the posterior midgut of 3-day-old *esg-GAL4*,*UAS-GFP/+* flies were set at 1. The midguts were stained with anti-Prospero or DAPI, after which the number of enteroendocrine cells or enterocytes with large nuclei was assessed. For quantitative analysis, the images were processed in Adobe Photoshop and the number of enteroendocrine cells or enterocytes with large nuclei was counted in 0.12 × 0.02 cm or 0.06 × 0.02 cm area of the posterior midgut, respectively. The results are expressed as the mean ± SE values of 27–35 adults. Two asterisks represent *P* < 0.001, and a single asterisk represents *P* < 0.05 as compared to each control.

We first assessed the effect of PVF2 levels on the proliferative activity of the adult midgut. To determine whether PVF2 levels could influence DNA synthesis in the adult midgut, BrdU incorporation level was monitored. The *Pvf2^c06947^* mutant flies showed reduced BrdU-labeled midgut cells, relative to those of 3-day-old wild-type flies, and the BrdU incorporation levels did not increase with age in the *Pvf2^c06947^* mutant flies (Supplementary Fig. S6A,a–c). Furthermore, *Pvf2^c06947^* flies did not exhibit a paraquat-induced increase in BrdU-labeled midgut cells, which was observed in control wild-type flies (Supplementary Fig. S6A, d–e). We then assessed DNA synthesis using the BrdU incorporation assay in the midguts of *esg-GAL4,UAS-GFP*/*UAS-PVF2-RNAi* flies with or without paraquat treatment. Whereas the paraquat-treated midguts of *esg-GAL4,UAS-GFP*/+ flies showed a marked increase in the number of BrdU-labeled cells, no significant changes were observed in paraquat-treated midguts of *esg-GAL4,UAS-GFP*/*UAS-PVF2-RNAi* flies (Supplementary Fig. S6B). Furthermore, *esg-GAL4*,*UAS-GFP*/*UAS-PVF2* flies showed marked increases in the number of BrdU-labeled cells relative to *esg-GAL4*,*UAS-GFP*/+ flies (Supplementary Fig. S6C). We then assessed PCNA mRNA levels in 3-, 30- and 60-day-old *Pvf2^c06947^* flies, and in paraquat-treated *Pvf2^c06947^* flies. The levels of PCNA mRNA did not change in the *Pvf2^c06947^* flies with age or with paraquat treatment, in contrast to wild-type flies (Supplementary Fig. S1B). Consistent with this finding, the levels of PCNA mRNA in the midguts of *esg-GAL4*,*UAS-GFP*/*UAS-PVF2-RNAi* flies remained unchanged following paraquat treatment (Supplementary Fig. S1C). We also attempted to determine whether PVF2 overexpression using *esg-GAL4* affected PCNA gene expression by using flies carrying a *PCNA-lacZ* reporter fusion gene. *PCNA-lacZ* expression in the midguts of 5-day-old flies carrying both *UAS-PVF2* and *esg-GAL4* was markedly higher than that of the 5-day-old flies carrying only *esg-GAL4* (Supplementary Fig. S7). Next, we examined the role of PVF2 levels on the number of proliferating cells in the midgut in response to aging and oxidative stress by using anti-phospho-histone H3 antibody staining. The number of phospho-histone H3-positive cells in *Pvf2^c06947^* flies did not increase with age or in response to paraquat treatment relative to negative controls (Fig. 6A-a; Supplementary Table S1). The midguts of 3-day-old *esg-GAL4*,*UAS-GFP*/*UAS-PVF2-RNAi* flies showed lower numbers of phospho-histone H3-positive cells relative to the midguts of *esg-GAL4*,*UAS-GFP*/+ flies (Supplementary Table S1). Furthermore, we did not observe a significant increase in the numbers of phospho-histone H3-positive cells in the aged or paraquat-treated *esg-GAL4,UAS-GFP*/*UAS-PVF2-RNAi* flies, in contrast to the increased number of proliferating cells in the aged or paraquat-treated *esg-GAL4,UAS-GFP*/+ flies (Fig. 6A-a; Supplementary Table S1). We next assessed the effects of PVF2 or PVR overexpression on the number of proliferating cells within the adult midgut. The number of phospho-histone H3-labeled cells increased significantly in *esg-GAL4,UAS-GFP*/*UAS-PVF2* flies (8.5 ± 1.0 pHH3-positive cells) and *esg-GAL4*,*UAS-GFP*/*UAS-PVR* flies (9.9 ± 0.8 pHH3-positive cells) relative to *esg-GAL4*,*UAS-GFP*/+ flies (4.0 ± 0.4 pHH3-positive cells) (Fig. 6A-b; Supplementary Table S1). Taken together, these results indicated that increased PVF2 levels were associated with age- and oxidative stress-induced increases in the number of proliferating cells in the adult midgut.

We then assessed the effect of PVF2 levels on the number and activity of ISCs and progenitor cells in the adult midgut. First, to determine whether PVF2 was also involved in the age- and oxidative stress-related modulation in the number of ISCs, we examined changes in the number of Delta-positive cells relative to PVF2 levels. In contrast to the observed increases in Delta-positive cells with aging and in response to oxidative stress in wild-type flies, the number of Delta-positive cells observed in *Pvf2^c06947^* flies did not increase with age or paraquat treatment (Fig. 6B; Supplementary Fig. S8 and Table S3). We also evaluated the number of Delta-positive cells in the adult midgut of *esg-GAL4,UAS-GFP*/*UAS-PVF2-RNAi* flies with age or paraquat treatment. In contrast to the dramatic increase in the number of Delta-positive cells in the midgut of the *esg-GAL4,UAS-GFP*/+ flies with age or paraquat treatment, the number of Delta-positive cells in *esg-GAL4,UAS-GFP*/*UAS-PVF2-RNAi* flies remained unchanged following aging or paraquat treatment (Fig. 6B; Supplementary Table S3).

To determine the effect of PVF2 overexpression on the number of *esg*-positive cells, we stained the midguts of the 3-day-old *esg-GAL4,UAS-GFP/+* and *esg-GAL4,UAS-GFP*/*UAS-PVF2* flies with anti-GFP and anti-Armadillo antibodies. Overexpression of PVF2 by *esg-GAL4* led to a significant increase (up to 2.2-fold) in *esg*-positive small cells within the adult midgut relative to control flies (Fig. 6C (b,j); Supplementary Table S2). We determined the effects of *esg-GAL4*-driven PVR overexpression and PVF2 knockdown on the number of ISCs and/or enteroblasts. PVR overexpression led to a significant increase (up to 2.4-fold) in the number of *esg*-positive cells relative to control flies (Fig. 6C (c,j); Supplementary Table S2). In contrast to the significant increase in *esg*-positive cells induced by PVF2 or PVR overexpression, flies bearing *esg-GAL4* and *UAS-PVF2-RNAi* showed a substantial reduction in the number of *esg*-positive cells (data not shown). Next, we determined whether PVF2 overexpression could modulate the Delta-positive cell population in the adult midgut. Interestingly, PVF2 overexpression under the control of the *esg-GAL4* driver was shown to increase the number of Delta-positive cells (up to 2.4-fold), within the adult midgut relative to control flies (Fig. 6C (e,j); Supplementary Table S3). PVR overexpression under the control of the *esg-GAL4* driver also led to a significant increase (up to 3.2-fold) in the number of Delta-positive cells within the adult midgut relative to control flies (Fig. 6C (f,j); Supplementary Table S3). Furthermore, the midguts from *Su(H)GBE-lacZ* reporter flies bearing *UAS-PVF2* and *esg-GAL4* or *UAS-PVR* and *esg-GAL4* showed a significant increase (up to 1.8-fold in both cases) in the number of Su(H)-positive cells relative to controls (Fig. 6C (g–i,j); Supplementary Table S4). The ratios of *esg*-positive/Delta-positive cells and Delta-positive/Su(H)-positive cells were also determined (Table 1). These results indicate that overexpression of PVF2 or PVR in ISCs and enteroblasts led to an increase in the ISC cell population in a manner similar to that associated with aging or oxidative stress.

Furthermore, whereas the number of enteroendocrine cells increased 1.4-fold or 1.7-fold, differentiated enterocytes with large nuclei decreased by 0.7-fold or 0.6-fold in the adult midgut of *esg-GAL4*/*UAS-PVF2* or *esg-GAL4*/*UAS-PVR* flies, respectively, relative to control *esg-GAL4*/+ flies (Fig. 6C-k). This result indicated that PVF2 levels play a key role in the differentiation of enterocytes. Collectively, these results demonstrated that PVF2 levels in the midgut contributed significantly to the increase in ISC populations and to the decrease in the number of differentiated enterocytes in the adult *Drosophila* midgut that occur with aging and in response to oxidative stress.

## Discussion

In the adult small intestinal epithelium, self-renewal depends on a highly regulated process that includes the proliferation of multipotent stem cells and the differentiation of unipotent daughter cells or progenitor cells (reviewed in Potten *et al*., 1997; Weissman, 2000). Knowledge of the underlying regulatory mechanisms associated with age-related changes in ISCs is vitally important to our understanding of cancer development, as cancer is considered an age-related disease (reviewed in Krtolica & Campisi, 2002; Balducci & Ershler, 2005). The phenomenon of intrinsic aging of gut epithelial stem cells has been clearly established; however, many questions remain unanswered. The adult *Drosophila* midgut is an excellent model system for studying stem cell biology (Micchelli & Perrimon, 2006; Ohlstein & Spradling, 2006, 2007).

In the present study, we assessed age-related changes in the adult *Drosophila* midgut. We initially detected age-associated increases in the proliferative activity of the adult *Drosophila* midgut via an increase in BrdU-labeled cells, PCNA expression and the number of proliferating cells. Our results are consistent with previous reports showing an increase in proliferative activity in the intestine with advancing age in other organisms (Holt & Yeh, 1988, 1989; reviewed in Jaszewski *et al*., 1999; Xiao *et al*., 2001). Taken together, these findings suggest that age-related increases in intestinal proliferative activity may be conserved from *Drosophila* to mammals, and that *Drosophila* is an excellent model system for the identification of regulatory factors that can modulate the self-renewal of stem cells during aging.

Herein, we assessed age-associated changes in the number of ISCs and progenitor cells in the adult fly midgut. Interestingly, we identified an age-related increase in the number of ISCs in the *Drosophila* midgut. We determined the ratio of *esg*-positive/Delta-positive cells and Delta-positive/Su(H)-positive cells (Table 1), which demonstrated that the ISC population in the midgut increases with age. If stem cells undergo symmetric cell divisions to generate two daughter stem cells, the stem cell pool may expand (Dingli *et al*., 2007). Our data suggest that symmetric stem cell divisions in the *Drosophila* midgut increase with age, and provide evidence that these divisions are affected significantly by the aging process. However, our analysis of *esg*-positive and Su(H)-positive cells demonstrated an age-related increase in the number of enteroblasts, indicating that asymmetric stem cell divisions in the midgut may also increase with age. We then determined whether the increase in enteroblasts led to an increase in the number of enteroendocrine cells or enterocytes. We found that the number of enteroendocrine cells in the midgut increased in an age-dependent manner (Fig. 2K; Supplementary Table S5), whereas the number of differentiated enterocytes with large nuclei decreased in an age-dependent manner (Fig. 2K; Supplementary Table S5). These data suggested that enterocyte differentiation may be disrupted within the aged gut and that the age-related changes may occur in the lineage from the potential stem cells to the terminally differentiated cells of gut epithelium.

In the present study, we used paraquat treatment and flies carrying the catalase mutation to demonstrate that age-related changes in the midgut can be mimicked by conditions that induce an oxidative stress response (Fig. 3). Our data suggest that the accumulation of oxidative damage may be a primary factor underlying the age-related changes in stem cells in the gut. Many studies have shown that intracellular ROS levels increase with age (reviewed in Dröge, 2003). Furthermore, ROS induces the activation of a variety of signaling cascades, including mitogen-activated protein kinase (MAPK) and nuclear factor-kappa B (NF-κB) pathways (reviewed in Torres & Forman, 2003; Gloire *et al*., 2006), which contribute to tumorigenesis via their ability to induce cell proliferation (reviewed in Esposito *et al*., 2004). Activation of p38 MAPK in response to increasing ROS levels has also been reported to limit the lifespan of hematopoietic stem cells *in vivo* (Ito *et al*., 2006). Our data also suggest that antioxidant enzymes, such as catalase, play a critical role with respect to the functional activity of stem cells. Cells from a hypocatalasemic patient expressing approximately 25% of normal *catalase* levels showed an accumulation of hydrogen peroxide and exhibited age-associated pathological changes (Wood *et al*., 2006). Hypocatalasemic individuals are known to experience a premature onset of age-related diseases, including tumors (reviewed in Terlecky *et al*., 2006).

Here, we established *Pvf2-lacZ* transgenic flies and demonstrated that *Pvf2-lacZ* is specifically expressed in ISCs and enteroblasts of the adult midgut (Fig. 4). These data suggest that *Pvf2-lacZ* is a useful specific marker of ISCs and enteroblasts of the adult midgut. To confirm that endogenous *Pvf2* is expressed specifically in ISCs and enteroblasts, anti-Pvf2 immunostaining will be required. Importantly, we observed that the levels of *Pvf2* mRNA in the adult fly midgut increased with age and in response to oxidative stress induced by paraquat treatment and when catalase function was compromised in *Cat^n1^* mutant flies (Fig. 5). These results provide support for a previous report describing an increase in VEGF production in response to increased ROS levels in human cancer cells (Liu *et al*., 2006; Zhou *et al*., 2007). At present, our data showing an age-related increase in the number of *Pvf2-lacZ*-positive cells in the midgut indicated that an age- or oxidative stress-related increase in PVF2 levels in the midgut may be due primarily to an increase in the number of PVF2-producing cells in the midgut. Therefore, whether *Pvf2* mRNA level per the ISC or enteroblast is regulated at transcriptional level is unclear.

Herein, we discovered that PVF2 is a critical factor involved in age- and oxidative stress-related changes in the number and activity of stem and progenitor cells in the *Drosophila* midgut. We first determined that the adult fly midgut failed to exhibit age- and oxidative stress-related increases in BrdU-labeled cells, PCNA expression and the number of proliferating cells when PVF2 function was inhibited in ISCs and enteroblasts that expressed either the *Pvf2^c06947^* mutant allele or a transgene for PVF2 knockdown (Fig. 6A-a; Supplementary Figs S1 and S6). In addition, we showed that PVF2 or PVR overexpression driven by *esg-GAL4* in ISCs and enteroblasts led to an increase in BrdU-labeled cells, increased PCNA expression and an increase in the number of proliferating cells in the adult midgut, in a manner similar to that associated with aging and oxidative stress (Fig. 6A-b; Supplementary Figs S6 and S7). These data suggested that PVF2 levels played a significant role in the regulation of cell proliferation in the midgut. We demonstrated that PVF2 levels regulated the number of ISCs via an increase in the number of Delta-positive cells by comparing the ratios of *esg*-positive/Delta-positive cells and Delta-positive/Su(H)-positive cells in flies that overexpressed PVF2 or PVR in ISCs and enteroblasts (Fig. 6C, Table 1; Supplementary Table S3). We showed that PVF2 or PVR overexpression led to a decrease in the number of differentiated enterocytes with large nuclei in the adult midgut (Fig. 6C-k; Supplementary Table S5); this result indicated that PVF2 levels can impact enterocyte differentiation. In mammals, VEGF has been shown to regulate hematopoietic stem cell proliferation via an autocrine loop mechanism (reviewed in Gerber & Ferrara, 2003; Fragoso *et al*., 2007). The VEGF receptor flt-1 is expressed on intestinal epithelial cells (Siafakas *et al*., 1999); however, the role of VEGF in intestinal cells is not known. Our data suggest a role for VEGF/VEGFR signaling in the differentiation of enterocytes.

In this study, we investigated the effect of aging on the number and activity of stem and progenitor cells in the *Drosophila* midgut. We demonstrated that age-related changes in the number and activity of stem and progenitor cells of the adult midgut can be mimicked by oxidative stress and that the level of PVF2, a PDGF/VEGF-like growth factor, is a critical modulator of the number and activity of stem and progenitor cells in the fly midgut, indicating a novel role for PVF2/PVR signaling. Furthermore, we established *Pvf2-lacZ* transgenic flies that will be useful in future studies aimed at the identification of the upstream or downstream signaling pathways inherent to the regulation of ISC self-renewal.

## Experimental procedures

### Plasmid construction

To construct the plasmid pPvf2-lacZ, the promoter region of the *Pvf2* gene was PCR-cloned with *Drosophila* genomic DNA. Amplified 1501-bp fragments including the *Pvf2* promoter region (–1220 to +281 with respect to the transcription initiation site) were then ligated into the *Kpn* I site of pCaSpeR-AUG-β-gal harboring the P-element, and sequenced.

### Establishment of transgenic fly and fly stock

Fly stocks were maintained at 25 °C on standard food under an ∼12 h/12 h light/dark cycle. The food consisted of 79.2% water, 1% agar, 7% cornmeal, 2% yeast, 10% sucrose, 0.3% bokinin and 0.5% propionic acid. To avoid larval overpopulation in all vials, 50–60 adult flies per vial were transferred to new food vials every 2–3 days for a period of 50–60 days or longer. In order to establish transgenic flies including *Pvf2-lacZ*, P-element-mediated germ-line transformation was conducted as described previously (Rubin & Spradling, 1982). The pPvf2-lacZ construct described above was co-injected with the pπ25.7wc helper vector (Karess & Rubin, 1984) into *w^1118^* embryos, transformants were selected and lines with two-copy inserts were established. Five independent lines were generated with pPvf2-lacZ. Transgenic lines including the fusion gene evidenced the same *lacZ* expression pattern. *Su(H)GBE-lacZ* was kindly provided by Sarah Bray (Furriols & Bray, 2001). *UAS-PVR* was generously provided by Pernille Rørth (Duchek *et al*., 2001). *Pvf2^c06947^*, *UAS-PVF2* (Cho *et al*., 2002) and *Cat^n1^* were acquired from the Bloomington Drosophila Stock Center. *esg-GAL4* and *UAS-PVF2-RNAi* was kindly provided by the Drosophila Genetic Resource Center and the National Institute of Genetics, respectively. *Oregon-R* was used as wild-type. The *esg-GAL4,UAS-GFP*/*UAS-PVF2;Su(H)GBE-lacZ*/+ flies were obtained from a cross of the *UAS-PVF2*/*UAS-PVF2;Su(H)GBE-lacZ*/TM6 males (derived from a cross of the *UAS-PVF2*/*UAS-PVF2*;TM6/+ males to the +/SM1;*Su(*H**)*GBE-lacZ*/Pre females which were derived from a cross of the *Pm*/SM1;Pre/TM3 males to the +/+;*Su*(*H)GBE-lacZ*/TM3 females) to the *esg-GAL4,UAS-GFP*/*CyO* females. The *esg-GAL4,UAS-GFP*/*UAS-PVR*;*Su(H)GBE-lacZ*/+ flies were obtained from a cross of the *UAS-PVR*/*UAS-PVR*;*Su(H)GBE-lacZ*/Pre males (derived from a cross of the *UAS-PVR*/*UAS-PVR*;Pre/+ males to the +/SM1;*Su*(*H*)*GBE-lacZ*/TM3 females which were derived from a cross of the *Pm*/SM1;Pre/TM3 males to the +/+;*Su*(*H)GBE-lacZ*/TM3 females) to the *esg-GAL4,UAS-GFP*/*CyO* females. The *esg-GAL4*,*UAS-GFP*/+;*Su(H)GBE-lacZ*/+ flies from a cross of the *Su(H)GBE-lacZ*/TM3 males to the *esg-GAL4,UAS-GFP*/*CyO* females were used as a control. All experiments were conducted and evidenced similar results in both females and males. The results described in this present study were obtained from the females.

### Oligonucleotides

All oligonucleotides were chemically synthesized. The primers for the *Pvf2* promoter region were 5′-ggcggtaccATGCATGCCTATGATGACGA-3′ and 3′-CGTAAAGTGTTGAAGCGCATccatggcgg-5′. Oligonucleotide primers for RT-PCR were as follows: ribosomal protein 49 (*rp49*): 5′-GACAACAGAGTCGGTCGC-3′ and 3′-TTCAAGGACCACGTGTTG-5′; *Pvf2*: 5′-ACGACAATCATCTCAGCT-3′ and 3′-GTGAAGTTCCTGAAGTCA-5′; PCNA: 5′-CCAACAACGAGGACAATGTG-3′ and 3′-GACTGTAAACGGACAGCGAT-5′; *Delta*: 5′-CAGTTCCGGCAGCTTTGAGT-3′ and 3′-GTGCTTAGGGTAGGTCAAGG-5′.

### RT-PCR

Total RNA from the adult midguts was isolated with Trizol Reagent (Molecular Research Center, Cincinnati, OH, USA) in accordance with the protocols recommended by the manufacturer. In brief, adult midguts were dissected, chloroform was added (Sigma, St. Louis, MO, USA) and then the samples were repeatedly centrifuged at 19 326 × ***g*** at 4 °C for 15 min. The supernatant was moved to a new microtube, isopropanol was added, and the samples were incubated at 25 °C for 15 min, centrifuged repeatedly at 19 326 × ***g*** at 4 °C for 15 min, washed with 70% EtOH, and dried. cDNAs from the prepared mRNA extracts were synthesized. Denatured mRNA with M-MLV-RT buffer, 2.5 mm dNTP, oligo dT, 100 mm DTT and M-MLV-reverse transcriptase (Promega, Madison, WI, USA) was incubated at 42 °C for 1 h. All the samples were processed for 30 PCR cycles (95 °C for 30 s; 52 °C for 30 s; 72 °C for 30 s), with a final extension step at 72 °C for 5 min The RT-PCR products were analyzed on 2% agarose gels stained with ethidium bromide.

### Immunochemistry

The intact adult guts were dissected, fixed at room temperature for 1 h in 4% formaldehyde (Sigma), washed with PBT [0.1% Triton X-100 in phosphate-buffered saline (PBS)], and incubated overnight with primary antibody at 4 °C. After washing and blocking [2% bovine serum albumin (BSA) in PBT], the samples were incubated for 1 h with secondary antibodies at 25 °C, washed in PBT, mounted with Vectashield (Vector Laboratories, Burlingame, CA, USA), and analyzed using a Zeiss Axioskop 2plus microscope (Carl Zeiss Inc., Gottingen, Germany). For the quantitative analysis of *esg*-, Delta- and Su(H)-positive cells and enterocytes and enteroendocrine cells, images were processed in Photoshop (Adobe Systems, San Jose, CA, USA). The numbers of *esg*-, Delta- and Su(H)-positive cells and enterocytes were counted in 0.06 × 0.02 cm area and the number of enteroendocrine cells were counted in 0.12 × 0.02 cm area of the posterior midgut.

For immunohistostaining of the midgut, the midguts were dissected, fixed at 25 °C for 2 h in 4% paraformaldehyde (Sigma) and washed in PBS. After being frozen in tissue-freezing medium (Leica Microsystems, Wetzlar, Germany), sections were cut (8 µm thick) on a cryostat (LEICA CM 1850, Leica Microsystems) and incubated overnight with primary antibody at 4 °C. After washing and blocking (2% BSA in PBT), the samples were incubated for 1 h with secondary antibodies at 25 °C, washed in PBT, mounted with Vectashield (Vector Laboratories), and analyzed using a Zeiss Axioskop 2plus microscope.

### BrdU labeling

BrdU staining was conducted via standard methods and modified as follows (Micchelli & Perrimon, 2006). The adult flies were cultured on standard media vials augmented with 200 µL of 6 mg mL^−1^ BrdU (Sigma) plus 20% sucrose. Flies were subsequently cultured at 25 °C and transferred to new media every 2 days for between 6 and 10 days to achieve maximal labeling. Adult flies were dissected in Ringer's solution. The entire guts were removed and fixed in ethanol : acetic acid (3 : 1) for 2 min, and were hydrolyzed for 10 min with 2 N HCl, then incubated with primary antibody overnight at 4 °C. Primary antibody was removed and the samples were washed in PBT. The samples were incubated for 1 h with secondary antibodies and DAPI, washed in PBT and mounted with Vectashield (Vector Laboratories).

### Antisera

The following primary antibodies diluted in PBT were used in these experiments: rabbit anti-β-gal (Cappel, Solon, OH, USA) 1 : 500; rabbit anti-phospho-histone H3 (Upstate, Charlottesville, VA, USA) 1 : 500; mouse anti-Armadillo, mouse anti-Prospero, mouse anti-Delta and mouse anti-BrdU (Developmental Studies Hybridoma Bank, Iowa City, IA, USA) 1 : 20; mouse anti-GFP, rabbit anti-GFP (Molecular Probes, Eugene, OR, USA) 1 : 500. The following secondary antibodies diluted in PBT + 2% BSA were used: goat anti-rabbit FITC (Cappel) 1 : 300; goat anti-rabbit Cy3 (Jackson ImmunoResearch, West Grove, PA, USA) 1 : 300; goat anti-mouse FITC (Jackson ImmunoResearch) 1 : 300; goat anti-mouse Cy3 (Jackson ImmunoResearch) 1 : 300; DAPI (Molecular Probes) 1 : 1000.

### Quantitative measurement of β-galactosidase activity in extracts

Quantitative measurement of β-galactosidase activity in extracts prepared from the midguts was conducted as described previously (Fridell & Searles, 1992). The adult midguts were dissected, homogenized in 100 µL of ice-cold assay buffer [50 mm potassium phosphate (pH 7.5) and 1 mm MgCl_2_] and centrifuged at 14 240 × ***g*** at 4 °C for 10 min For the assay, supernatant was added to 200 µL of assay buffer containing 1 mm CPRG substrate (Roche, Basel, Switzerland) and incubated at 37 °C in darkness. The substrate conversion was measured at 574 nm at 0.5, 1, 1.5 and 2 h after the addition of the extract. During this period, the rate of color development was linear. The β-galactosidase activity was defined as absorbance units per milligram per hour of protein. The protein amounts were determined with a BCA protein kit (Sigma).

### Paraquat assay and measurement of ROS generation

Flies were dry-starved at 25 °C for 4 h and exposed to 10 mm paraquat (Sigma) in 1% sucrose media for 16 h. Total ROS generation was measured using the previously described method (Trifunovic *et al*., 2005) and modified slightly as follows. The adult midguts were dissected, homogenized in homogenizing buffer (50 mm potassium phosphate buffer, pH 7.4), and centrifuged at 14 240 × ***g*** at 4 °C for 10 min. Twenty-five mm DCFDA (Invitrogen, Carlsbad, CA, USA) was added to the extracts and changes in fluorescence intensity were determined with a Multilabel counter (PerkinElmer VICTOR3 V, PerkinElmer Life and Analytical Sciences, Waltham, MA, USA) with an excitation wavelength of 485 nm and an emission wavelength of 530 nm for 1 h. The fluorescence intensities were normalized to the protein amounts determined by the BCA protein kit (Sigma).

### X-gal staining

The adult guts were dissected on ice and fixed for 10 min with 1% glutaraldehyde (Sigma) in 1× PBS. The samples were then washed in 1× PBS for 1 h, and stained with 0.2% X-gal (USB, Cleveland, OH, USA) in staining buffer containing 6.1 mm K_4_Fe(CN)_6_, 6.1 mm K_3_Fe(CN)_6_, 1 mm MgCl_2_, 150 mm NaCl, 10 mm Na_2_HPO_4_ and 10 mm NaH_2_PO_4_ in the dark at 25 °C during overnight.

### Statistical analyses

Quantified data are expressed as the mean ± SE values. Significance testing was conducted via Student's *t*-test.

## Supplementary material

Fig. S1 Effects of aging and oxidative stress on proliferating cell nuclear antigen (PCNA) mRNA levels in the adult midgut of wild-type, Pvf2^c06947^ mutant and PVF2 knockdown flies. (A) The effects of aging and oxidative stress on PCNA mRNA levels in the adult midgut of wild-type flies. Total RNA was isolated from the midguts of 3-, 30- and 60-day-old wild-type flies, 5-dayold wild-type flies exposed to 10 mm paraquat in 1% sucrose or 1% sucrose media as controls for 16 h, 5-day-old wild-type and Cat^n1^ mutant flies. Then, cDNAs were synthesized and PCNA mRNA levels were analyzed via RT-PCR. (B) The effects of aging and oxidative stress on PCNA mRNA levels in the adult midgut of Pvf2^c06947^ mutant flies. PCNA mRNA levels in the midguts of 3-, 30- and 60-day-old Pvf2^c06947^ flies, 5-day-old Pvf2^c06947^ flies exposed to 10 mm paraquat in 1% sucrose or 1% sucrose media as controls for 16 h were assessed. (C) The effect of oxidative stress on PCNA mRNA levels in the adult midgut of PVF2 knockdown flies. PCNA mRNA levels in the midguts of 5-day-old esg-GAL4,UAS-GFP/+ and esg-GAL4,UAS-GFP/UAS-PVF2-RNAi flies incubated in media with or without 10 mm paraquat for 16 h were assessed. All rp49 was utilized as a loading control.

Fig. S2 Increased reactive oxygen species (ROS) levels in the adult midgut of Cat^n1^ mutant flies. Relative ROS levels were assessed in the adult midguts of wild-type and Cat^n1^ mutant flies as described previously. Two asterisks represent P < 0.005, and a single asterisk represents P < 0.01 as compared to each control.

Fig. S3 Effects of oxidative stress on 5-bromodeoxyuridine BrdU) incorporation levels and the number of proliferating cells in the adult midgut. (A) Effects of paraquat treatment and the Cat^n1^ allele on the BrdU incorporation levels. The 12-day-old wild-type flies fed on BrdU for 10 days were dry-starved for 4 h at 25 °C, 1% sucrose media as control (a) or exposed to 10 mm paraquat in 1% sucrose media for 16 h (b) and the adult midguts were stained with anti-BrdU. The 12-day-old Cat^n1^ mutant flies (c) fed on BrdU for 10 days were dry-starved for 4 h at 25 °C and the adult midguts were stained with anti-BrdU. Overlay (DAPI, blue; anti-BrdU, red). Magnification is ×400 in each panel (a–C). a′–Cc′: Enlarged image of each panel. Scale bar, 20 μm. (B) Effects of paraquat treatment and the Cat^n1^ allele on the number of phospho-histone H3-positive cells. The midguts of 5-day-old wild-type flies exposed to 10 mm paraquat in 1% sucrose (b) or 1% sucrose media as controls (a) for 16 h and 5-day-old Cat^n1^ mutant flies (c) were labeled with anti-phosphohistone H3 (arrow). Overlay (DAPI, blue; anti-phospho-histone H3, red). Scale bar, 20 μm.

Fig. S4 Effects of oxidative stress on the number of ISCs and progenitor cells in the adult midgut. (a–Cc) Effects of paraquat treatment and the Cat^n1^ allele on the number of Delta-positive cells in the adult midgut of wild-type flies. The midguts of 5-day-old wild-type flies exposed to 10 mm paraquat in 1% sucrose (b) or 1% sucrose media as control (a) for 16 h, and 5-day-old Cat^n1^ mutant flies (c) were stained with anti-Delta (arrow). Overlay (DAPI, blue; anti-Delta, red). (d–Cf) Effects of paraquat treatment and the Cat^n1^ allele on the numbers of Delta-positive cells and Su(H)-positive cells in the adult midgut of Su(H)GBE-lacZ flies. The midguts of 5-day-old Su(H)GBE-lacZ flies exposed to 10 mm paraquat in 1% sucrose (e) or 1% sucrose media as control (d) for 16 h, and 5-day-old Su(H)GBElacZ/ atn1 flies (f) were stained with anti-β-gal (arrowhead) and anti-Delta (arrow). Overlay (DAPI, blue; anti-β-gal, green; anti-Delta, red). All scale bar, 20 μm.

Fig. S5 Expression of the *Pvf2-lacZ* fusion gene in the guts of embryo and larvae. *Pvf2-lacZ* expression was detected in the guts of embryo at late stage with anti-β-gal (a, arrow), and in the guts of the third instar larvae by X-gal staining (b).

Fig. S6 Effects of PVF2 level on BrdU incorporation levels in the adult midgut. (A) Effects of aging and paraquat treatment on BrdU incorporation levels in the adult midgut of Pvf2^c06947^ mutant flies. (a–Cc) The 2-, 30- and 50-day-old Pvf2^c06947^ flies were fed on BrdU for 10 days and the adult midguts were stained with anti-BrdU. (d–Ce) The 12-day-old Pvf2^c06947^ flies fed on BrdU for 10 days were dry-starved for 4 h at 25 °C, exposed to 10 mm paraquat in 1% sucrose media (e) or 1% sucrose media as control (d) for 16 h, and the adult midguts were stained with anti-BrdU. Magnification is ×400 in each panel. (B) Effect of paraquat treatment on BrdU incorporation levels in the adult midgut of *esg-GAL4,UAS-GFP/+* and *esg-GAL4,UAS-GFP/UAS-PVF2-RNAi* flies. The 2-day-old *esg-GAL4,UAS-GFP/+* (a, b) and *esg-GAL4,UAS-GFP/UAS-PVF2-RNAi* (c, d) flies were fed on BrdU for 8 days, exposed to 10 mm paraquat in 1% sucrose media (b, d) or 1% sucrose media as control (a, c) for 16 h, and the adult midguts were stained with anti-BrdU. Scale bar, 20 μm. (C) Effect of PVF2 overexpression under the control of *esg-GAL4* on BrdU incorporation levels in the adult midgut. The 2-day-old *esg-GAL4,UAS-GFP/+* (a) and *esg-GAL4,UAS-GFP/UAS-PVF2* (b) flies were fed on BrdU for 8 days and the adult midguts were stained with anti-BrdU. Scale bar, 20 μm. Overlay (DAPI, blue; anti-BrdU, red).

Fig. S7 Effect of PVF2 overexpression under the control of *esg-GAL4* on PCNA expression in the adult midgut. *PCNA-lacZ* expression was detected in the midguts of *esg-GAL4,UAS-GFP/+;PCNA-lacZ/+* (a) and *esg-GAL4,UAS-GFP/UAS-PVF2;PCNAlacZ/+* (b) flies by X-gal staining.

Fig. S8 Effects of aging and oxidative stress on Delta-positive cell populations in the adult midgut of Pvf2^c06947^ mutant flies. (a–c) The midguts of 3-, 30- and 60-day-old Pvf2^c06947^ flies were labeled with anti-Delta (arrow). (d–e) The midguts of 5-day-old Pvf2^c06947^ flies exposed to 10 mm paraquat in 1% sucrose (e) or 1% sucrose media as a control (d) for 16 h were stained with anti-Delta. Overlay (DAPI, blue; anti-Delta, red). Scale bar, 20 μ.

Table S1 The numbers of phospho-histone H3-positive cells in the adult midgut with age, ROS production and PVF2 level

Table S2 The numbers of esg-positive cells in the adult midgutwith age, ROS production and PVF2 level

Table S3 The numbers of Delta-positive cells in the adult midgut with age, ROS production and PVF2 level

Table S4 The numbers of Su(H)-positive cells in the adult midgut with age, ROS production and PVF2 level

Table S5 The numbers of enteroendocrine cells and enterocytes in the adult midgut with age, ROS production and PVF2 level

Table S6 The numbers of *Pvf2-lacZ*-positive cells and esgpositive cells in the adult midgut of *Pvf2-lacZ/esg-GAL4*, flies by ROS production

Table S7 The numbers of *Pvf2-lacZ*-positive cells and Deltapositive cells in the adult midgut of *Pvf2-lacZ* reporter flies with age and ROS production

Table S8 The percentages of adult flies with *esg-GAL4,UASGFP/UAS-PVF2*, *esg-GAL4,UAS-GFP/UAS-PVR* or *esg-GAL4*, *UAS-GFP/UAS-PVF2-RNAi*

This material is available as part of the online article from: http://www.blackwell-synergy.com/doi/abs/10.1111/j.1474-9726.2008.00380.x (This link will take you to the article abstract).
