# Supplementary material for: Age-related changes in Drosophila midgut are associated with PVF2, a PDGF/VEGF-like growth factor
Source: Aging Cell. 2008 Jun;7(3):318–34. doi: 10.1111/j.1474-9726.2008.00380.x (PMC2408640; doi:10.1111/j.1474-9726.2008.00380.x)

Supplementary Figure. S1

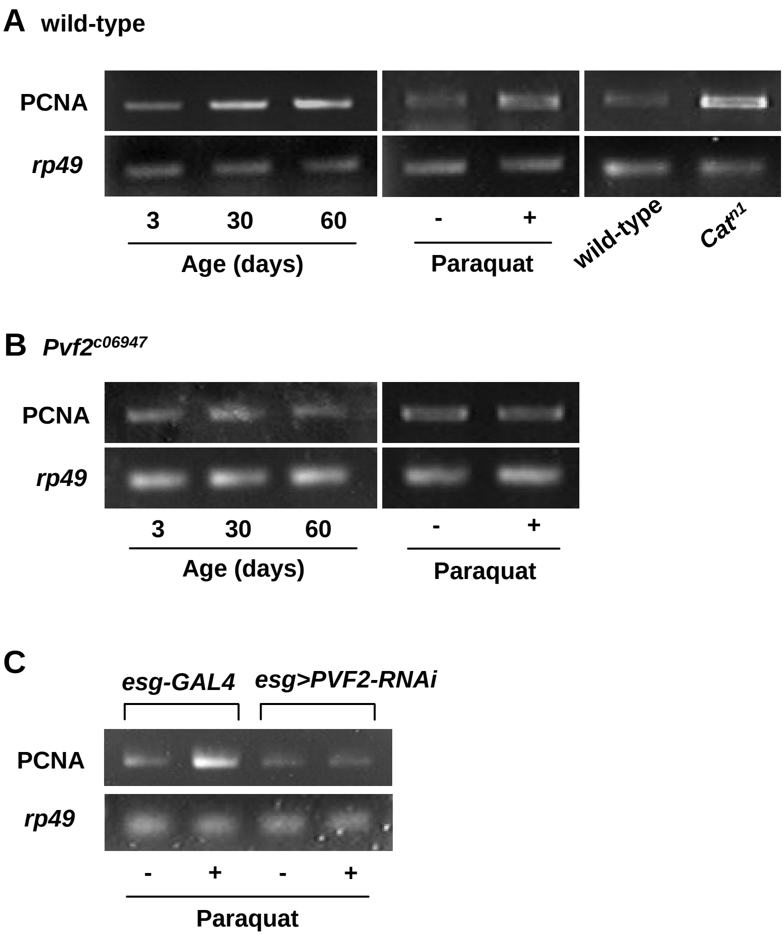

Supplementary Figure. S2

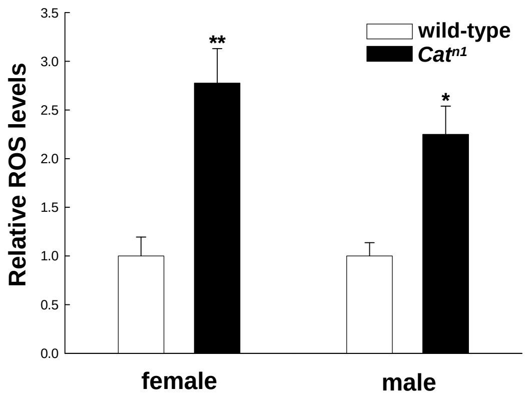

Supplementary Figure. S3

A

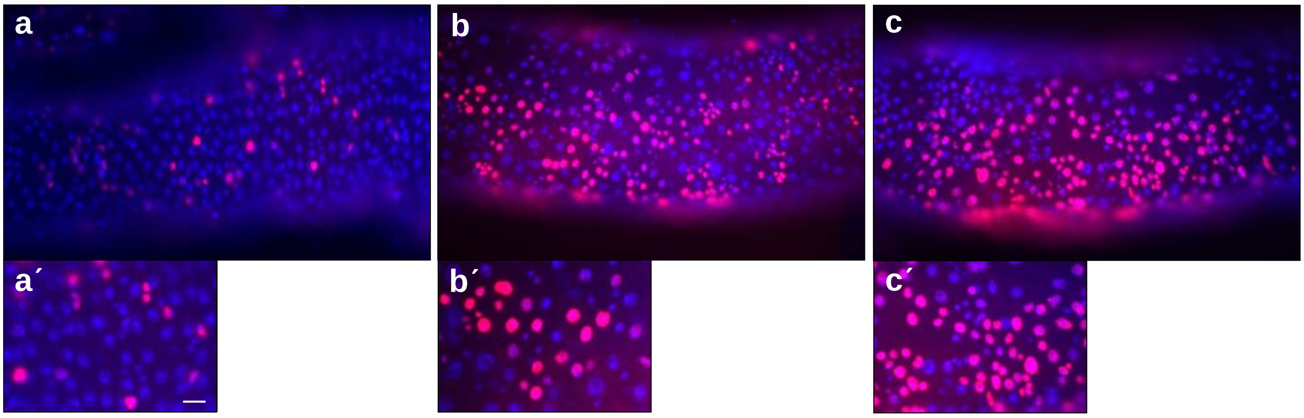

B

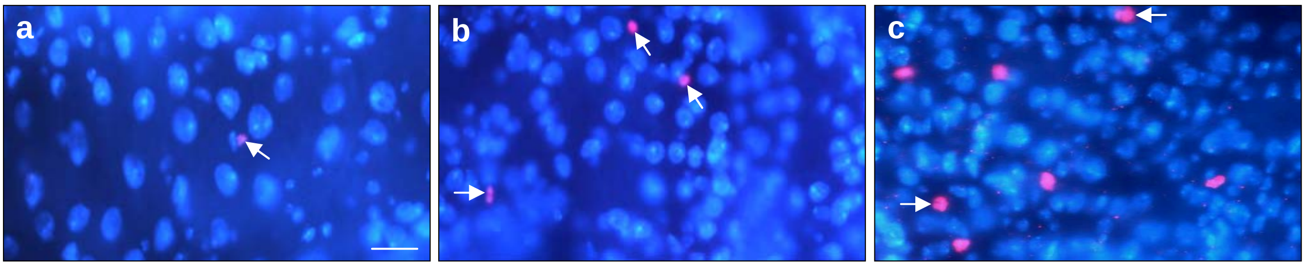

Supplementary Figure. S4

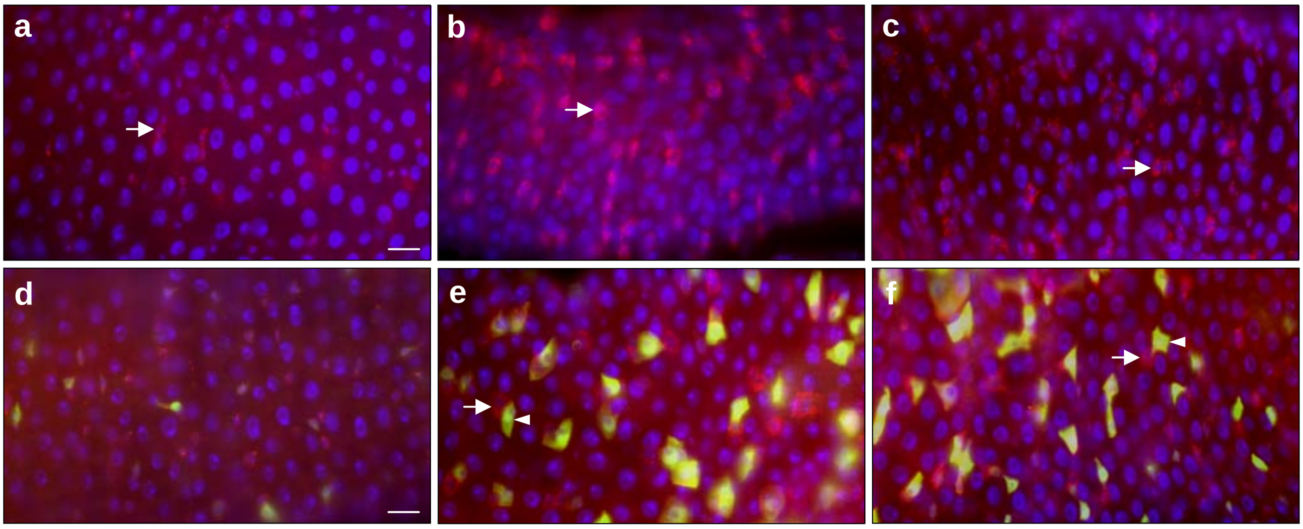

Supplementary Figure. S5

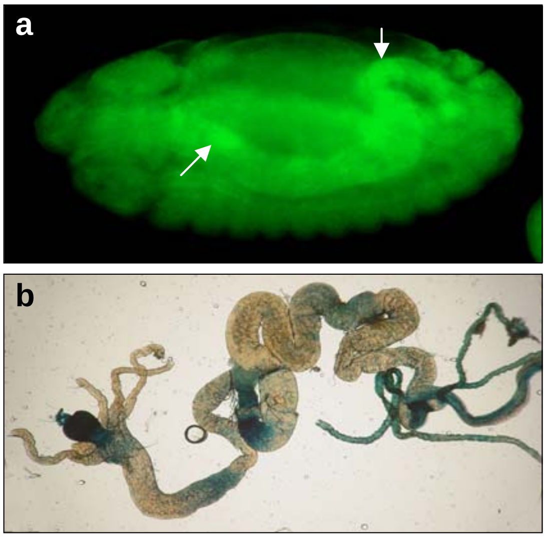

Supplementary Figure. S6

A

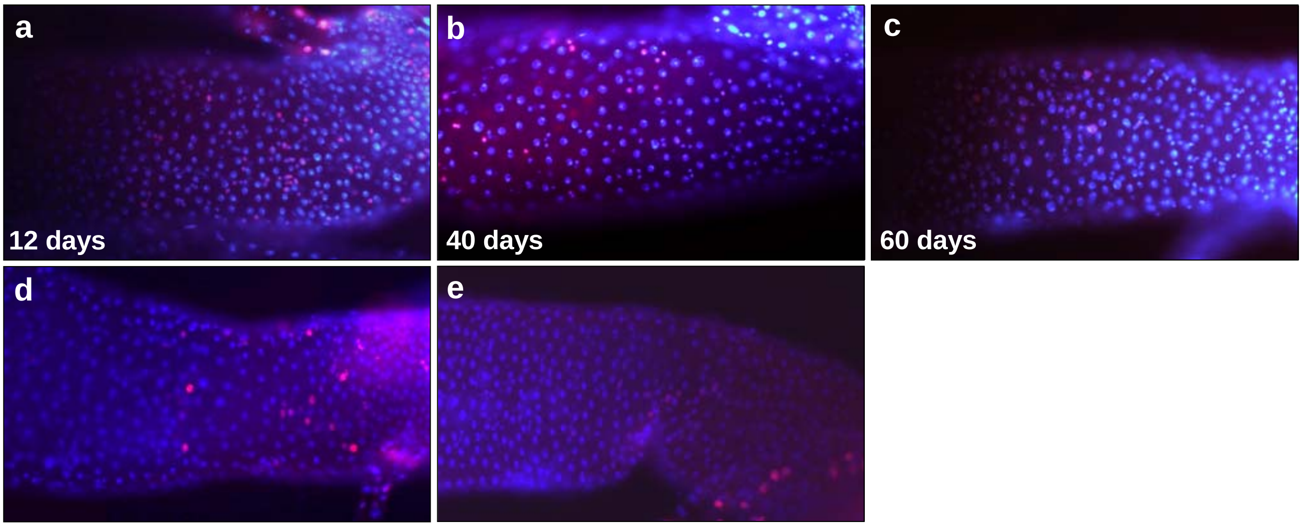

B

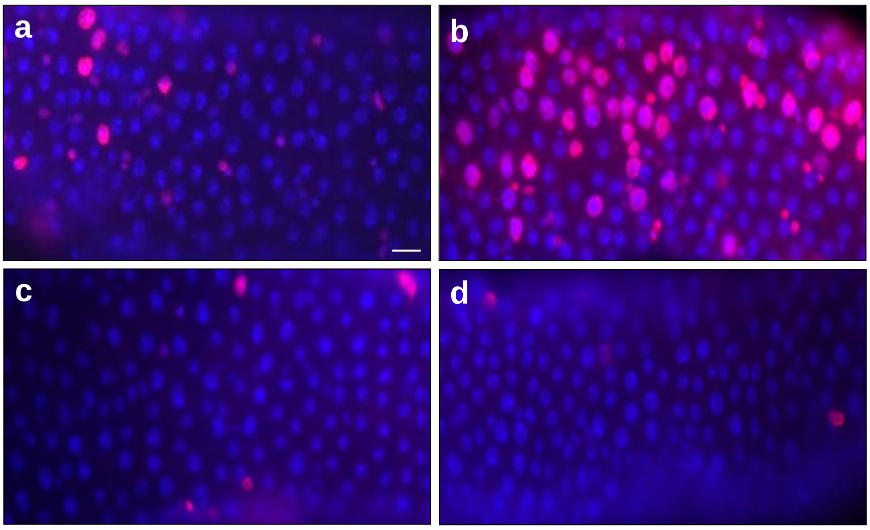

C

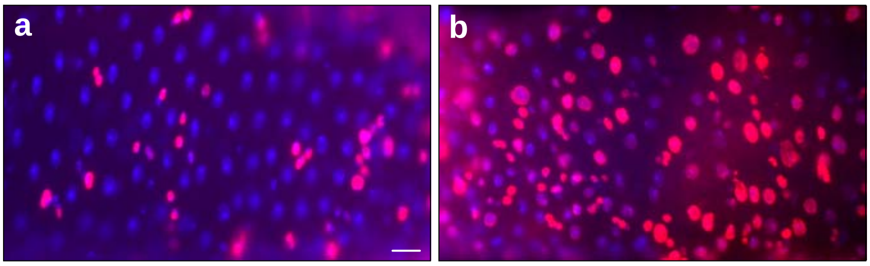

Supplementary Figure. S7

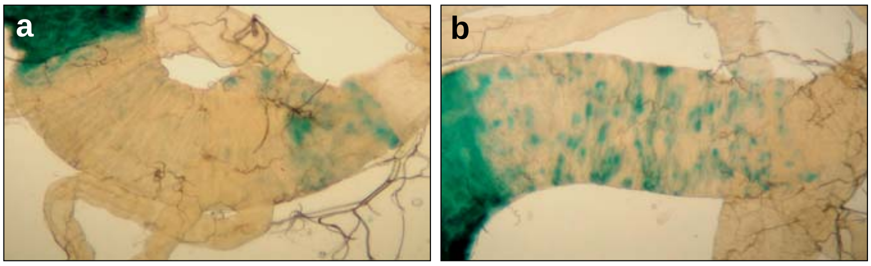

Supplementary Figure. S8

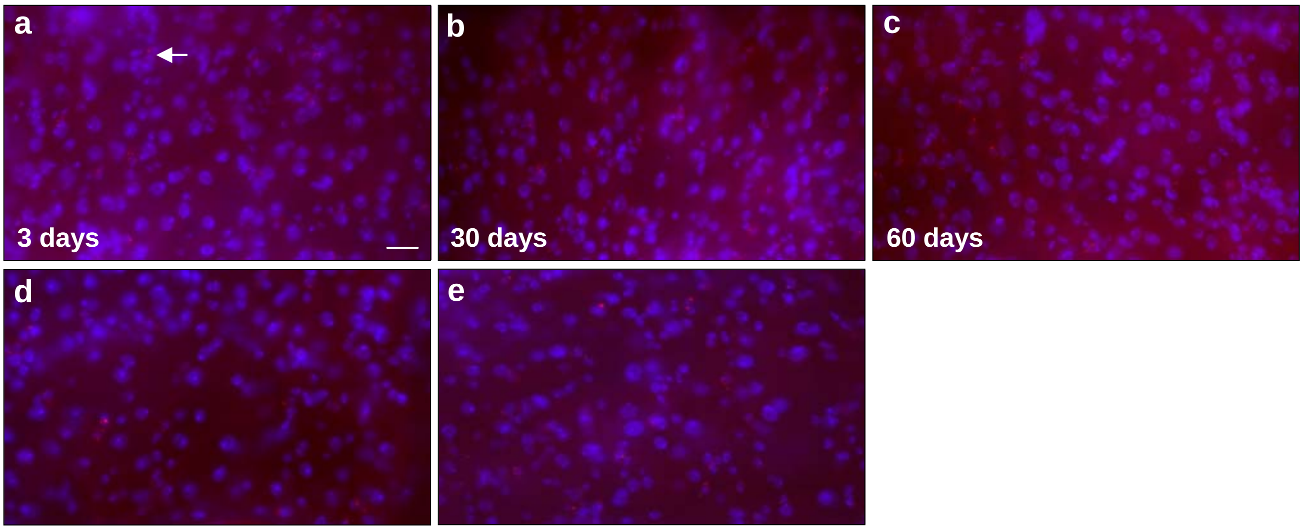

Supplement: Fig. S1 — Effects of aging and oxidative stress on proliferating cell nuclear antigen (PCNA) mRNA levels in the adult midgut of wild-type, Pvf2c06947 mutant and PVF2 knockdown flies. (A) The effects of aging and oxidative stress on PCNA mRNA levels in the adult midgut of wild-type flies. Total RNA was isolated from the midguts of 3-, 30- and 60-day-old wild-type flies, 5-dayold wild-type flies exposed to 10 mm paraquat in 1% sucrose or 1% sucrose media as controls for 16 h, 5-day-old wild-type and Catn1 mutant flies. Then, cDNAs were synthesized and PCNA mRNA levels were analyzed via RT-PCR. (B) The effects of aging and oxidative stress on PCNA mRNA levels in the adult midgut of Pvf2c06947 mutant flies. PCNA mRNA levels in the midguts of 3-, 30- and 60-day-old Pvf2c06947 flies, 5-day-old Pvf2c06947 flies exposed to 10 mm paraquat in 1% sucrose or 1% sucrose media as controls for 16 h were assessed. (C) The effect of oxidative stress on PCNA mRNA levels in the adult midgut of PVF2 knockdown flies. PCNA mRNA levels in the midguts of 5-day-old esg-GAL4,UAS-GFP/+ and esg-GAL4,UAS-GFP/UAS-PVF2-RNAi flies incubated in media with or without 10 mm paraquat for 16 h were assessed. All rp49 was utilized as a loading control. [file ace0007-0318-SD1.pdf]
