# Supplementary material for: Age-related changes in Drosophila midgut are associated with PVF2, a PDGF/VEGF-like growth factor
Source: Aging Cell. 2008 Jun;7(3):318–34. doi: 10.1111/j.1474-9726.2008.00380.x (PMC2408640; doi:10.1111/j.1474-9726.2008.00380.x)
Supplement: Table S1 — The numbers of phospho-histone H3-positive cells in the adult midgut with age, ROS production and PVF2 level [file ace0007-0318-SD9.doc]

**Supplementary Table**

**Table S1.** The numbers of phospho-histone H3-positive cells in the adult midgut with age, ROS production and PVF2 level

|  | **wild-type (*Oregon-R*)** | | | | | | | | |
| --- | --- | --- | --- | --- | --- | --- | --- | --- | --- |
|  | **Age (days)** | | | |  | **ROS production** | | | |
|  | **3** | **30** | **45** | **60** |  | **Con** | **PQ** | **Con** | ***Cat^n1^*** |
| Mean ± SE | 4.1 ± 0.6 | 7.4 ± 0.7 | 11.4 ± 1.1 | 12.8 ± 1.1 |  | 3.9 ± 0.4 | 9.0 ± 0.8 | 3.9 ± 0.5 | 17.6 ± 1.6 |
| n | 43 | 41 | 41 | 45 |  | 52 | 50 | 39 | 42 |
| Cell number | 178 | 303 | 466 | 577 |  | 200 | 450 | 151 | 739 |

|  | ***Pvf2^c06947^*** | | | | | | | | | | |
| --- | --- | --- | --- | --- | --- | --- | --- | --- | --- | --- | --- |
|  | **Age (days)** | | | | | |  | **ROS production** | | | |
|  |  | **3** | **30** | **45** | **60** |  |  |  | **Con** | **PQ** |  |
| Mean ± SE |  | 5.3 ± 0.7 | 5.7 ± 0.6 | 6.2 ± 0.6 | 5.0 ± 0.5 |  |  |  | 4.9 ± 0.7 | 4.5 ± 0.6 |  |
| n |  | 42 | 45 | 38 | 37 |  |  |  | 39 | 39 |  |
| Cell number |  | 223 | 252 | 236 | 183 |  |  |  | 190 | 176 |  |

|  | ***esg-GAL4*/+** | | | | | | | |  |  | ***esg-GAL4*/*UAS-PVF2-RNAi*** | | | | | | | |
| --- | --- | --- | --- | --- | --- | --- | --- | --- | --- | --- | --- | --- | --- | --- | --- | --- | --- | --- |
|  | **Age (days)** | | | | | | | |  |  | **Age (days)** | | | | | | | |
|  |  | **3** | | **30** | | **60** | |  |  |  |  | **3** | | **30** | | **60** | |  |
| Mean ± SE |  | 3.0 ± 0.5 | | 7.2 ± 0.4 | | 12.3 ± 0.9 | |  |  |  |  | 1.6 ± 0.2 | | 1.6 ± 0.2 | | 1.3 ± 0.2 | |  |
| n |  | 29 | | 30 | | 28 | |  |  |  |  | 28 | | 31 | | 30 | |  |
| Cell number |  | 86 | | 217 | | 343 | |  |  |  |  | 44 | | 50 | | 39 | |  |
|  | **ROS production** | | | | | | | |  |  | **ROS production** | | | | | | | |
|  |  | | **Con** | | **PQ** | |  | |  |  |  | | **Con** | | **PQ** | |  | |
| Mean ± SE |  | | 4.0 ± 0.6 | | 8.0 ± 0.8 | |  | |  |  |  | | 2.1 ± 0.3 | | 1.9 ± 0.3 | |  | |
| n |  | | 61 | | 53 | |  | |  |  |  | | 56 | | 58 | |  | |
| Cell number |  | | 243 | | 426 | |  | |  |  |  | | 118 | | 110 | |  | |

|  |  | **PVF2/PVR overexpression** | | |  |
| --- | --- | --- | --- | --- | --- |
|  |  | ***esg-GAL4*/+** | ***esg-GAL4*/*UAS-PVF2*** | ***esg-GAL4*/*UAS-PVR*** |  |
| Mean ± SE |  | 4.0 ± 0.4 | 8.5 ± 1.0 | 9.9 ± 0.8 |  |
| n |  | 53 | 52 | 56 |  |
| Cell number |  | 211 | 443 | 553 |  |

Phospho-histone H3-expressing cell numbers per adult midgut are expressed as the mean ± SE values of the observed total cell number (Cell number) of analyzed flies (n). Con; control, PQ; Paraquat.

**Table S2.** The numbers of *esg*-positive cells in the adult midgut with age, ROS production and PVF2 level

|  | ***esg-GAL4*** | | | | | | | | | |
| --- | --- | --- | --- | --- | --- | --- | --- | --- | --- | --- |
|  | **Age (days)** | | | | |  | **ROS production** | | | |
|  |  | **3** | **30** | **50** |  |  |  | **Con** | **PQ** |  |
| Mean ± SE |  | 57.5 ± 1.5 | 122.7 ± 4.4 | 147.3 ± 3.6 |  |  |  | 69.8 ± 3.1 | 149.7 ± 6.9 |  |
| n |  | 69 | 37 | 35 |  |  |  | 26 | 25 |  |
| Cell number |  | 3966 | 4541 | 5155 |  |  |  | 1816 | 3743 |  |

|  |  | **PVF2/PVR overexpression** | | |  |
| --- | --- | --- | --- | --- | --- |
|  |  | ***esg-GAL4*/+** | ***esg-GAL4*/*UAS-PVF2*** | ***esg-GAL4*/*UAS-PVR*** |  |
| Mean ± SE |  | 63.4 ± 2.6 | 136.5 ± 5.6 | 148.4 ± 5.8 |  |
| n |  | 40 | 35 | 37 |  |
| Cell number |  | 2537 | 4778 | 5491 |  |

The numbers of *esg*-positive cells are expressed as the mean ± SE values of the observed total cell number (Cell number) of analyzed flies (n). Con; control, PQ; Paraquat.

**Table S3.** The numbers of Delta-positive cells in the adult midgut with age, ROS production and PVF2 level

|  | **wild-type (*Oregon-R*)** | | | | | | | | |
| --- | --- | --- | --- | --- | --- | --- | --- | --- | --- |
|  | **Age (days)** | | |  |  | **ROS production** | | | |
|  | **3** | **30** | **60** |  |  | **Con** | **PQ** | **Con** | ***Cat^n1^*** |
| Mean ± SE | 19.1 ± 1.6 | 56.7 ± 4.1 | 77.6 ± 4.8 |  |  | 23.1 ± 1.3 | 79.7 ± 4.2 | 19.2 ± 1.8 | 84.0 ± 4.2 |
| n | 33 | 31 | 31 |  |  | 35 | 32 | 26 | 29 |
| Cell number | 629 | 1759 | 2406 |  |  | 808 | 2549 | 500 | 2437 |

|  | **wild-type (*esg-GAL4*)** | | | | | | | | | |
| --- | --- | --- | --- | --- | --- | --- | --- | --- | --- | --- |
|  | **Age (days)** | | | | |  | **ROS production** | | | |
|  |  | **3** | **30** | **50** |  |  |  | **Con** | **PQ** |  |
| Mean ± SE |  | 22.4 ± 1.5 | 61.9 ± 4.6 | 79.7 ± 7.1 |  |  |  | 20.2 ± 2.2 | 71.8 ± 5.0 |  |
| n |  | 43 | 34 | 26 |  |  |  | 21 | 19 |  |
| Cell number |  | 962 | 2103 | 2071 |  |  |  | 424 | 1364 |  |

|  | **wild-type (*Su(H)GBE-lacZ*)** | | | | | | | | |
| --- | --- | --- | --- | --- | --- | --- | --- | --- | --- |
|  | **Age (days)** | | |  |  | **ROS production** | | | |
|  | **3** | **30** | **60** |  |  | **Con** | **PQ** | **Con** | ***Cat^n1^*** |
| Mean ± SE | 21.3 ± 3.5 | 70.3 ± 4.1 | 86.2 ± 4.6 |  |  | 25.4 ± 3.2 | 86.2 ± 3.5 | 22.3 ± 2.2 | 82.8 ± 6.8 |
| n | 20 | 21 | 19 |  |  | 27 | 31 | 18 | 16 |
| Cell number | 425 | 1476 | 1638 |  |  | 685 | 2673 | 402 | 1324 |

|  | ***Pvf2^c06947^*** | | | | | | | | | |
| --- | --- | --- | --- | --- | --- | --- | --- | --- | --- | --- |
|  | **Age (days)** | | | | |  | **ROS production** | | | |
|  |  | **3** | **30** | **60** |  |  |  | **Con** | **PQ** |  |
| Mean ± SE |  | 27.0 ± 2.4 | 29.5 ± 2.1 | 28.8 ± 2.6 |  |  |  | 26.1 ± 1.7 | 29.2 ± 2.4 |  |
| n |  | 27 | 32 | 28 |  |  |  | 30 | 26 |  |
| Cell number |  | 730 | 944 | 806 |  |  |  | 784 | 758 |  |

|  | ***esg-GAL4*/+** | | | | | | | |  |  | ***esg-GAL4*/*UAS-PVF2-RNAi*** | | | | | | | |
| --- | --- | --- | --- | --- | --- | --- | --- | --- | --- | --- | --- | --- | --- | --- | --- | --- | --- | --- |
|  | **Age (days)** | | | | | | | |  |  | **Age (days)** | | | | | | | |
|  |  | **3** | | **30** | | **60** | |  |  |  |  | **3** | | **30** | | **60** | |  |
| Mean ± SE |  | 18.8 ± 1.8 | | 58.8 ± 5.0 | | 78.5 ± 7.2 | |  |  |  |  | 7.8 ± 1.5 | | 7.5 ± 1.0 | | 8.7 ± 1.1 | |  |
| n |  | 20 | | 18 | | 17 | |  |  |  |  | 17 | | 20 | | 19 | |  |
| Cell number |  | 375 | | 1058 | | 1335 | |  |  |  |  | 133 | | 149 | | 165 | |  |
|  | **ROS production** | | | | | | | |  |  | **ROS production** | | | | | | | |
|  |  | | **Con** | | **PQ** | |  | |  |  |  | | **Con** | | **PQ** | |  | |
| Mean ± SE |  | | 20.3 ± 2.2 | | 76.7 ± 4.5 | |  | |  |  |  | | 8.3 ± 1.2 | | 9.7 ± 1.3 | |  | |
| n |  | | 31 | | 32 | |  | |  |  |  | | 28 | | 30 | |  | |
| Cell number |  | | 630 | | 2455 | |  | |  |  |  | | 231 | | 292 | |  | |

|  |  | **PVF2/PVR overexpression** | | |  |
| --- | --- | --- | --- | --- | --- |
|  |  | ***esg-GAL4*/+** | ***esg-GAL4*/*UAS-PVF2*** | ***esg-GAL4*/*UAS-PVR*** |  |
| Mean ± SE |  | 21.4 ± 1.7 | 51.3 ± 2.8 | 69.4 ± 3.0 |  |
| n |  | 27 | 25 | 46 |  |
| Cell number |  | 577 | 1283 | 3192 |  |

The numbers of Delta-positive cells are expressed as the mean ± SE values of the observed total cell number (Cell number) of analyzed flies (n). Con; control, PQ; Paraquat.

**Table S4.** The numbers of Su(H)-positive cells in the adult midgut with age, ROS production and PVF2 level

|  | **wild-type (*Su(H)GBE-lacZ*)** | | | | | | | | |
| --- | --- | --- | --- | --- | --- | --- | --- | --- | --- |
|  | **Age (days)** | | |  |  | **ROS production** | | | |
|  | **3** | **30** | **60** |  |  | **Con** | **PQ** | **Con** | ***Cat^n1^*** |
| Mean ± SE | 70.2 ± 3.4 | 112.5 ±3.4 | 140.1±5.1 |  |  | 62.2 ± 3.8 | 118.2± 7.4 | 65.1 ± 4.4 | 123.4 ± 7.6 |
| n | 32 | 30 | 27 |  |  | 31 | 29 | 27 | 29 |
| Cell number | 2247 | 3376 | 3782 |  |  | 1928 | 3429 | 1758 | 3578 |

|  |  | **PVF2/PVR overexpression** | | |  |
| --- | --- | --- | --- | --- | --- |
|  |  | ***esg-GAL4*/+** | ***esg-GAL4*/*UAS-PVF2*** | ***esg-GAL4*/*UAS-PVR*** |  |
| Mean ± SE |  | 60.0 ± 4.3 | 105.1 ± 4.3 | 109.9 ± 5.3 |  |
| n |  | 30 | 25 | .36 |  |
| Cell number |  | 1801 | 2627 | 3956 |  |

The numbers of Su(H)-positive cells are expressed as the mean ± SE values of the observed total cell number (Cell number) of analyzed flies (n). Con; control, PQ; Paraquat.

**Table S5.** The numbers of enteroendocrine cells and enterocytes in the adult midgut with age, ROS production and PVF2 level

|  |  | **wild-type (*Oregon-R*)** | | | | | | |
| --- | --- | --- | --- | --- | --- | --- | --- | --- |
|  |  | **Age (days)** | | |  |  | **ROS production** | |
| **Cells** |  | **3** | **30** | **60** |  |  | **Con** | ***Cat^n1^*** |
| ee cells | Mean ± SE | 53.9 ± 2.7 | 106.8 ± 2.3 | 128.3 ± 3.7 |  |  | 68.1 ± 3.6 | 151.3 ± 8.2 |
|  | n | 29 | 28 | 30 |  |  | 36 | 40 |
|  | Cell number | 1564 | 2990 | 3848 |  |  | 2450 | 6050 |
| ECs | Mean ± SE | 238.7 ± 6.0 | 109.1 ± 5.7 | 81.8 ± 3.8 |  |  | 242.6 ± 7.5 | 170.2 ± 5.2 |
|  | n | 50 | 34 | 36 |  |  | 34 | 31 |
|  | Cell number | 11933 | 3709 | 2946 |  |  | 8249 | 5276 |

|  |  | **PVF2/PVR overexpression** | | | | |
| --- | --- | --- | --- | --- | --- | --- |
| **Cells** |  |  | ***esg-GAL4*/+** | ***esg-GAL4*/*UAS-PVF2*** | ***esg-GAL4*/*UAS-PVR*** |  |
| ee cells | Mean ± SE |  | 52.7 ± 3.1 | 71.1 ± 6.3 | 91.8 ± 4.6 |  |
|  | n |  | 35 | 28 | 31 |  |
|  | Cell number |  | 1845 | 1991 | 2847 |  |
| ECs | Mean ± SE |  | 236.9 ± 8.1 | 161.0 ± 5.9 | 139.7 ± 5.1 |  |
|  | n |  | 33 | 29 | 27 |  |
|  | Cell number |  | 7817 | 4668 | 3771 |  |

The numbers of enteroendocrine (ee) cells and enterocytes (ECs) with large nuclei were expressed as the mean ± SE values of the observed total cell number (Cell number) of analyzed flies (n). Con; control.

**Table S6. The numbers of *Pvf2-lacZ*-positive cells and *esg*-positive cells in the adult midgut of *Pvf2-lacZ*/*esg-GAL4* flies by ROS production**

|  |  |  | ***Pvf2-lacZ*/*esg-GAL4*** | |  |
| --- | --- | --- | --- | --- | --- |
|  |  |  | **ROS production** | |  |
| **Cells** |  |  | **Con** | **PQ** |  |
| *esg*+ | Mean ± SE |  | 58.6 ± 4.2 | 120.7 ± 7.7 |  |
|  | n |  | 15 | 17 |  |
|  | Cell number |  | 879 | 2051 |  |
| β-gal+ | Mean ± SE |  | 57.4 ± 4.2 | 119.4 ± 7.9 |  |
|  | n |  | 15 | 17 |  |
|  | Cell number |  | 861 | 2030 |  |
|  | Ratio of β-gal+/*esg*+ |  | 0.98 | 0.99 |  |

The 5-day-old *Pvf2-lacZ*/*esg-GAL4* flies exposed to 10 mM paraquat in 1 % sucrose or 1 % sucrose media as controls for 16 h were labeled with anti-GFP and anti-β-gal. The numbers of *esg*-positive cells and β-gal-positive cells were expressed as the mean ± SE values of the observed total cell number (Cell number) of analyzed flies (n). Con; control, PQ; Paraquat.

**Table S7.** The numbers of *Pvf2-lacZ*-positive cells and Delta-positive cells in the adult midgut of *Pvf2-lacZ* reporter flies with age and ROS production

|  | | ***Pvf2-lacZ*** | | | | | | |
| --- | --- | --- | --- | --- | --- | --- | --- | --- |
|  |  | **Age (days)** | | |  |  | **ROS production** | |
| **Cells** |  | **3** | **30** | **60** |  |  | **Con** | **PQ** |
| Delta+ | Mean ± SE | 21.6 ± 2.3 | 55.1 ± 2.7 | 70.5 ± 4.0 |  |  | 25.4 ± 1.9 | 88.0 ± 3.5 |
|  | n | 33 | 35 | 40 |  |  | 32 | 27 |
|  | Cell number | 714 | 1927 | 2820 |  |  | 812 | 2377 |
| β-gal + | Mean ± SE | 60.1 ± 2.6 | 117.0 ± 4.1 | 141.8 ± 5.1 |  |  | 67.6 ± 3.4 | 139.8 ± 6.3 |
|  | n | 31 | 34 | 38 |  |  | 31 | 30 |
|  | Cell number | 1863 | 3979 | 5387 |  |  | 2095 | 4193 |
|  | Ratio of β-gal+/Delta+ | 2.78 | 2.12 | 2.01 |  |  | 2.66 | 1.59 |

The numbers of Delta-positive cells and β-gal-positive cells were expressed as the mean ± SE values of the observed total cell number (Cell number) of analyzed flies (n). Con; control, PQ; Paraquat.

**Table S8.** The percentages of adult flies with *esg-GAL4,UAS-GFP*/*UAS-PVF2*, *esg-GAL4,UAS-GFP*/*UAS-PVR* or *esg-GAL4,UAS-GFP*/*UAS-PVF2-RNAi*

| **line** | ***CyO*/*UAS-PVF2*** | ***esg-GAL4*/*UAS-PVF2*** |
| --- | --- | --- |
| % | 90.7 | 9.3 |
| n | 1121 | 115 |
| **line** | ***CyO*/*UAS-PVR*** | ***esg-GAL4*/*UAS-PVR*** |
| % | 87.6 | 12.4 |
| n | 1894 | 269 |
| **line** | ***CyO*/*UAS-PVF2-RNAi* and *CyO*/*esg-GAL4*** | ***esg-GAL4*/*UAS-PVF2-RNAi*** |
| % | 71.0 | 29.0 |
| n | 1628 | 667 |

The indicated number of total flies (n) was monitored.
